# A predictive mechanochemical modeling framework for the deformation and remodeling of the nuclear lamina

**DOI:** 10.64898/2026.02.19.706840

**Published:** 2026-03-16

**Authors:** Emmet A. Francis, Einollah Sarikhani, Hamed Naghsh-Nilchi, Zeinab Jahed, Padmini Rangamani

**Affiliations:** 1 Department of Pharmacology, University of California San Diego, La Jolla, CA, USA; 2 Aiiso Yufeng Li Family Department of Chemical and Nano Engineering, University of California San Diego, La Jolla, CA, USA; 3 Department of Mechanical and Aerospace Engineering, University of California San Diego, La Jolla, CA, USA

**Keywords:** nuclear mechanics, lamin A/C, nanopillars, YAP/TAZ, systems biophysics, hyperelasticity, reaction-transport, cell signaling, nuclear envelope rupture, mechanomedicine, Biophysics, Computational Biology

## Abstract

Nuclear envelope stretch and rupture are common to cell spreading and migration in a variety of microenvironments, leading to marked changes in nucleocytoplasmic transport. Predicting cell response to different mechanochemical cues that are transmitted to the nucleus remains an open problem in the field of mechanomedicine. We developed a predictive modeling framework to examine how nuclear deformation on substrates with different nanotopographies influences nucleocytoplasmic transport and rearrangement of the nuclear lamina. Using the finite element method, we simulated nuclear compression by the perinuclear actin cap on substrates with arrays of nanopillars, modeling the nuclear envelope as a nonlinear elastic structure and coupling deformations to a biochemical model of lamin remodeling and nucleocytoplasmic transport. These simulations predicted regions of high nuclear envelope stretch adjacent to cell-nanopillar contacts, leading to maximized nuclear envelope tension on small nanopillars spaced by 4-5 microns. We then considered the effects on nuclear transport of YAP and TAZ and found that increased nuclear compression led to YAP/TAZ nuclear localization in agreement with previous experiments. Furthermore, the simulated force load per lamin was maximized on nanopillar substrates with high nuclear stretch. The magnitude of this load was modulated by the rate of actin cap assembly and the overall expression level of lamin A/C – decreasing lamin content in the nuclear envelope led to a higher likelihood of rupture. We validated this prediction in subsequent experiments with lamin-depleted U2OS cells, establishing the central importance of lamin transport and microenvironment nanotopography to nuclear mechanotransduction.

## Significance

Cell nuclei commonly experience large strains, but existing computational models do not explain the coupling between such deformations and molecular transport. Here, we present a modeling framework that includes the mechanics of nuclear deformations and the reaction-transport of molecules within the cytoplasm, nuclear envelope, and nuclear interior. As a well-controlled setup for comparing experiments and simulations, we consider nuclear indentations exhibited by cells on nanopillar substrates. Our simulations recapitulate measurements of nuclear YAP/TAZ localization from the literature and predict that low-lamin cells experience higher force loads at the nuclear envelope. We validate this prediction experimentally, showing that lamin-depleted cells are more likely to exhibit nuclear rupture. Overall, our framework presents opportunities to predict nuclear mechanoadaptation to different microenvironments.

## 3 Introduction

Cells respond to external biochemical and mechanical cues via shape changes and induction of intracellular signaling events. Changes in cell shape also affect the organization of intracellular structures such as the endoplasmic reticulum or mitochondria, as evidenced by dynamic measurements of organelle localization on substrates with different nanoscale features [1, 2]. The response of the nucleus is especially dramatic, as nuclear deformation is required for cell navigation through complex microenvironments [3-6]. In the extreme case of extravasation, immune cells or cancer cells must squeeze their nuclei between endothelial cells when migrating from blood vessels into surrounding tissue [7, 8]. Dissecting the forces required for organelle deformation and the implications for cellular state remains a pressing topic in the emerging field of mechanomedicine [9, 10].

Nuclear deformation acts as a key decision-making point in mechanotransduction by modulating nucleocytoplasmic transport. For instance, stretching of nuclear pore complexes (NPCs) in the nuclear envelope (NE) [11-13] and/or volumetric compression [14] have been shown to accelerate nuclear entry of YAP/TAZ and other molecules such as TWIST1. Furthermore, nuclear stretch leads to NE rupture (NER) in cells squeezing through small channels [3, 15, 16] or spreading on nanopillar substrates [17, 18]. Intermediate filaments known as lamins make up a significant portion of the layer beneath the NE double lipid bilayer and control NE stretch via interactions with the cytoskeleton mediated by the Linker of the Nucleoskeleton and Cytoskeleton (LINC) complex [19-21]. A-type lamins (lamin A/C) are particularly important determinants of nuclear rigidity [22-24] and the likelihood of NER [13, 16, 25]. The importance of a robust NE is clear from the many health problems associated with laminopathies, diseases defined by mutations in the gene for lamin A/C (LMNA) [26, 27]. Accordingly, it is crucially important to refine our quantitative understanding of how lamin content and organization regulate nuclear mechanotransduction.

**Figure 1:**
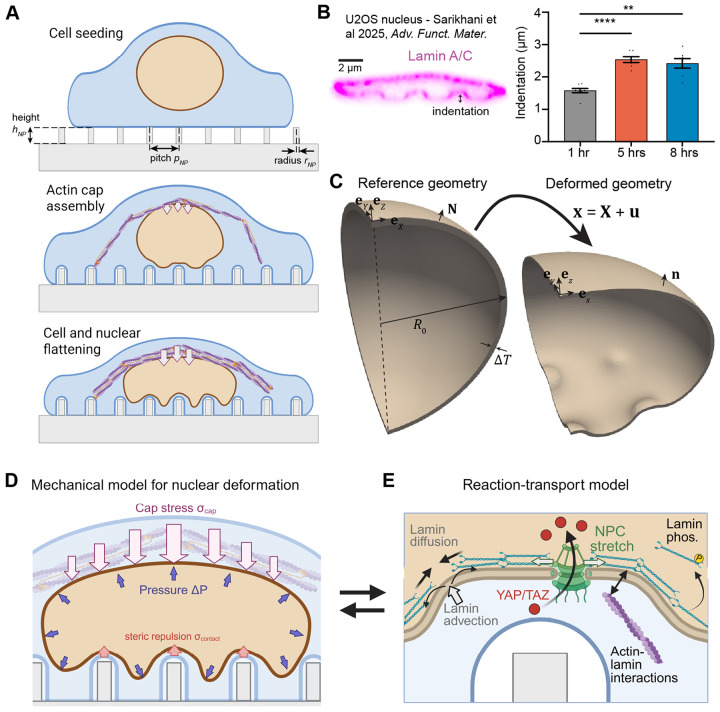
Modeling framework to study large nuclear deformations on nanopillar substrates. A) Schematic showing stages of cell adhesion and nuclear compression in cells on nanopillar substrates. Perinuclear actin cap assembly leads to cell and nuclear flattening over time. Variables describing nanopillar height $\left(h_{\text{NP}}\right)$, radius $\left(r_{\text{NP}}\right)$, and center-to-center spacing (pitch, $p_{\text{NP}}$) are defined in the upper schematic. B) Annotated scanning electron micrograph showing a U2OS nucleus on a nanopillar substrate from [17]. Reuse of content in panel B authorized by CC BY-NC-ND license, originally published as Fig 3B-C in [17]. C) Reference mesh and deformed geometry of NE shell utilized in our mechanical model. The reference mesh is a spherical shell with outer radius $R_{0}$ and thickness ΔT, deformed according to the displacement field u. Unit vectors denote coordinate directions ([X, Y, Z] in reference geometry, [x, y, z] in deformed geometry) and N and n denote the normal vectors associated with the reference and the deformed geometry, respectively. D) Hyperelastic mechanical model of the NE with applied stresses due to steric repulsion at nanopillar contact regions $\left(\sigma_{\text{contact}}\right)$, osmotic pressure (ΔP), and assembly of the actin cap $\left(\sigma_{\text{cap}}\right)$. E) Mixed-dimensional reaction-transport model for lamins, NPCs, and YAP/TAZ. This model includes the effects of dilution and aggregation due to advection of species in the NE, as well as diffusion of all species in the model. Arrows between panels D and E indicate bidirectional coupling via transport and stretch-sensitive NPC opening (left-to-right) and via lamin-dependent changes in nuclear stiffness (right-to-left). Schematics in A, D, and E created with BioRender.com.

Engineered substrates with nanoscale topographical features have offered a powerful experimental tool to probe the effects of nanotopography on the nucleus [17, 18, 28-30]. Features such as nanopillars or nanoneedles introduce well-controlled, reproducible nuclear indentations, facilitating the study of nucleoskeletal and cytoskeletal remodeling adjacent to induced NE curvature [17, 18, 28, 30] (Figure 1A-B). These experiments have shown that F-actin and lamin A/C localize adjacent to cell and nuclear indentations [18, 30, 31]. In addition to cytoskeletal activation due to curvature of the plasma membrane [31, 32], NE curvature modulates arrangement of the lamin nucleoskeleton [16, 30]. However, the mechanisms coupling NE deformation to lamin remodeling remain unclear.

Quantitative modeling of NE deformation has served as a robust and complementary approach to experimental techniques. Many studies in the literature are devoted to continuum models of NE deformation that describe the NE as an elastic shell with finite thickness [7, 33-37] or as an infinitesimally thin layer, analogous to the boundary of a liquid droplet [13, 38-40]. These continuum models facilitate predictions of nuclear morphology as a function of applied stress, e.g., due to micropipette aspiration [36], nuclear squeezing during migration [7, 38], or compression by the actin cytoskeleton [33, 37]. Although the mechanical properties of the nucleus have been characterized in some detail, coupling nuclear deformations to biochemical transport via advection and diffusion remains an open problem. Approaches to simulate mechanochemical coupling in the nucleus are required to answer several questions. For instance, how does rearrangement of lamins within the NE depend on the rate of deformation and on nanoscale topography of the microenvironment? Furthermore, in what ways do lamin remodeling and NPC stretch together determine changes in nucleocytoplasmic transport? Advances in three-dimensional simulations of reaction-transport systems and moving boundary problems in biology present opportunities to examine coupled mechanochemical problems [38, 41-43]. Thus, we are now well-positioned to answer questions about the effects of deformation on nucleocytoplasmic transport and lamin distribution.

Here, we used a coupled mechanochemical computational framework to examine the effects of nanotopography-induced nuclear deformation on nucleocytoplasmic transport and on lamin rearrangement. Building on our previous work [18], we simulated cell response to nanopillar substrates with varied height, radius, and spacing (Figure 1A). Our approach incorporated both mechanical deformation of the nucleus (Figure 1C-D) and reaction-transport of YAP/TAZ, NPCs, and lamins (Figure 1E). We hypothesized that nuclear compression and indentation lead to lamin A/C rearrangement within the NE, causing increased YAP/TAZ influx and increased likelihood of NER upon deformation. We compared our model predictions to correlations between YAP/TAZ nuclear localization and nuclear deformation in the literature, then leveraged our simulations of lamin dynamics against measurements of lamin A/C and NER in lamin-depleted U2OS cells. Thus, our model offers new insights into the complex interplay between NE mechanics and mechanotransduction signaling.

## 4 Results

### 4.1 Model development

Our modeling approach relied on two separate modules - a mechanical model describing deformation of the NE (Figure 1C-D) and a mixed-dimensional reaction-transport model describing evolution of NPCs, lamin A/C, and YAP/TAZ over time (Figure 1E). These two modules were bidirectionally coupled. Mechanical deformations drove changes in nuclear geometry, advective transport of signaling species, and stretch-mediated activation of NPCs. In turn, local lamin A/C density dictated the stiffness of the nuclear lamina. Below, we briefly summarize both modules. The governing equations are given in Section 6.1 and details on numerical implementation are provided in Section 6.2.

Our mechanical model treated the NE as a three-dimensional composite including two lipid bilayers and a thick layer of lamin filaments directly beneath (Figure 1D). The NE composite was approximated as an incompressible rubber-like hyperelastic material in line with previous quantitative models and experiments [33, 34, 37], allowing us to capture nonlinearities associated with large strains of elastic bodies [44, 45]. While the shell volume was incompressible, our model allowed the volume of the enclosed nucleoplasm to decrease dramatically during compression, corresponding to water loss through the semipermeable nuclear membrane [7, 33]. Similarly, the area of the inner and outer NE surfaces changed during our simulations, accommodated for by changes in the NE thickness. Biophysically, this corresponds to folding and unfolding of the nuclear membranes and lamina [13, 33, 40, 46]. In our simulations, we subjected an initially spherical NE shell to an osmotic pressure gradient [33] ΔP and a downward force due to the perinuclear actin cap $\sigma_{\text{cap}}$ (Figure 1D) [47, 48]. The lower region of the NE was subjected to a repulsive stress $\sigma_{\text{contact}}$ to capture the steric interactions with the actin layer between the NE and the plasma membrane adherent to the substrate [28] (Figure 1D). The magnitude of the perinuclear actin cap stress $\left(\sigma_{\text{cap}}\right)$ was varied to capture multiple states during actin cap assembly (Figure 1A) and, ultimately, to reproduce previous measurements of large-scale nuclear deformations observed in cells on nanopillar substrates (Figure 1B) [17, 28]. In all cases, we assumed that the nucleus was at mechanical equilibrium but the actin cap stress and/or the NE stiffness could vary in a time-dependent manner.

To capture the effects of nuclear deformation on nucleocytoplasmic transport and lamin A/C localization, we coupled the mechanical model to a biochemical reaction-transport model implemented in our recently developed software package, Spatial Modeling Algorithms for Reactions and Transport (SMART) [42]. In brief, this signaling model assumed preferential local actin assembly around inwardly curved regions of the cell membrane, leading to significant elevation above the global actin concentration throughout the cell. For the sake of simplicity, we did not explicitly consider actomyosin dynamics contributing to perinuclear cap assembly in this reaction-transport module, effectively treating actin within the perinuclear cap as a separate pool. Actin assembly facilitated activation of nuclear pore complexes (NPCs) within the NE, in cooperation with the underlying nuclear lamina (Figure 1E). We also tracked the advection and diffusion of lamins and NPCs with the NE during nuclear deformation. NPCs were assumed to diffuse slowly within the NE double bilayer and were activated by localization of F-actin and lamins and by mechanical stretch directly, as in our previous model [42]. Lamins were also assumed to effectively diffuse via lamin filament sliding within the network [16, 49], and they were advected with the inner surface of the NE, such that they were naturally diluted in regions of inward nuclear curvature as depicted in Figure 1E. We also considered the phosphorylation-regulated cycling of lamins between the nucleoplasm and NE (Figure 1E) [25, 50], wherein lamins were either retained at the NE in an actin-dependent manner or were phosphorylated and removed from the NE pool. Given these assumptions for lamin A/C and NPC dynamics, we predicted YAP/TAZ transport in and out of the nucleus, assuming that YAP/TAZ within the cytosol remained well-mixed over time.

We solved both the mechanical and the biochemical reaction-transport equations using the finite element method, as described in detail in Section 6.2. Below, we first consider the results from solving the mechanical model in isolation, before turning our attention to the coupled mechanochemical model of nuclear deformation and reaction-transport.

### 4.2 Nuclear envelope stretch and stress are maximized for moderate nanopillar pitch

We first examined the equilibrium conformation of the NE for increasing applied stresses on the upper region of the nucleus due to the perinuclear actin cap during its assembly. To probe the effects of microenvironment nanotopography on NE deformation, we simulated nuclear compression on different nanopillar substrates, with nanopillar radius $\left(r_{\mathrm{NP}}\right)$ 200 or 500 nm and height $\left(h_{\mathrm{NP}}\right)$ 1.5 or 3.0 μm. In each case, we examined the range of nuclear deformations and stress distributions exhibited as a function of nanopillar spacing (pitch, $p_{\mathrm{NP}}$), including deformation on a flat substrate as the control condition. We focused on NE stretch and NE tension as outputs because both are proposed predictors of accelerated nucleocytoplasmic transport [11, 12] and susceptibility to NER [16, 51].

**Figure 2:**
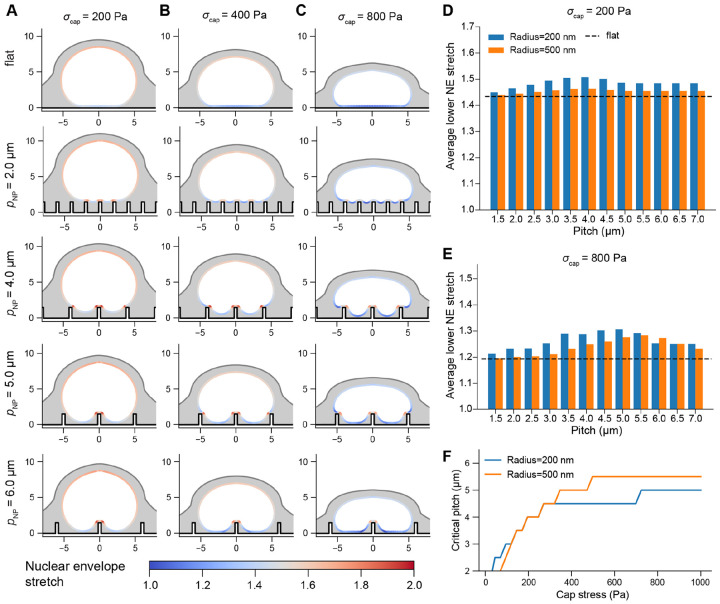
Nuclear envelope stretch exhibits a non-monotonic dependence on nanopillar pitch. A-C) Cross sections of equilibrium conformations of deformed nuclei on a flat substrate or substrates with $r_{\text{NP}}=200\;\text{nm}$, $h_{\text{NP}}=1.5\;\mu m$, and $p_{\text{NP}}=2\;\mu m$, 4μm, 5μm, or 6μm for cap stress $\left(\sigma_{\text{cap}}\right)$ of 200 Pa (A), 400 Pa (B), or 800 Pa (C). The computed stretch corresponds to stretch of the inner surface of the NE. D-E) Average stretch of the inner NE at the lower region of the nucleus as a function of nanopillar pitch for an applied cap stress of 200 Pa (D) or 800 Pa (E) on 200 nm or 500 nm radius nanopillars. Average stretch was computed over the elements of the inner NE with z values less than $z_{\text{center}}+0.05$, where $z_{\text{center}}$ is the z value associated with the point of the inner NE directly above the central nanopillar (Figure S1B). The dashed lines indicate stretch on a flat substrate. F) Critical pitch as a function of cap stress for $r_{\text{NP}}=200\;\text{nm}$ and $r_{\text{NP}}=500\;\text{nm}$.

We first assessed the effect of nanopillar size and spacing on indentation and stretch of the NE (Figure 2 and Movie 1). In agreement with previous experiments [28], nuclear indentation was minimal on low-pitch substrates – the nucleus rested on top of the pillar array even for high cap stresses, resulting in similar morphologies to a nucleus om a flat substrate (Figure 2A-C). Increasing the nanopillar pitch led to nuclear indentation accompanied by higher values of NE stretch adjacent to nanopillar contact regions (Figure 2A-C). The magnitude of this stretch averaged over the lower region of the NE increased up to some critical pitch value, past which this stretch declined (Figure 2D-E). We found that the value of critical pitch depended on the magnitude of the cap stress, which determined whether the nucleus contacted additional nanopillars. For instance, for $\sigma_{\text{cap}}=200$ Pa, the nucleus contacted only the central pillar when the pitch was greater than 4 μm (Figure 2A), so the maximum stretch was experienced for 3.5 to 4 μm pitch, where contacts with multiple nanopillars enhanced the average stretch over the lower NE. However, for higher cap stresses, the nucleus contacted additional nanopillars even they were spaced further apart, and thus the critical pitch increased accordingly (Figure 2E). This effect was observed for both 200 and 500 nm radius nanopillars (Figure 2D-E) and for both heights of 1.5 μm or 3.0 μm (Figure S2). On wider (500 nm radius) nanopillars, nuclei came into contact with the adjacent nanopillar at lower cap stresses (Figure S3), causing the relationship between NE stretch and pitch to shift rightward (Figure 2D-E). Overall, we found that the pitch associated with maximal NE stretch increased as a function of cap stress, with typical critical pitches reaching values of 4-5.5 μm for higher cap stresses (Figure 2F).

**Figure 3:**
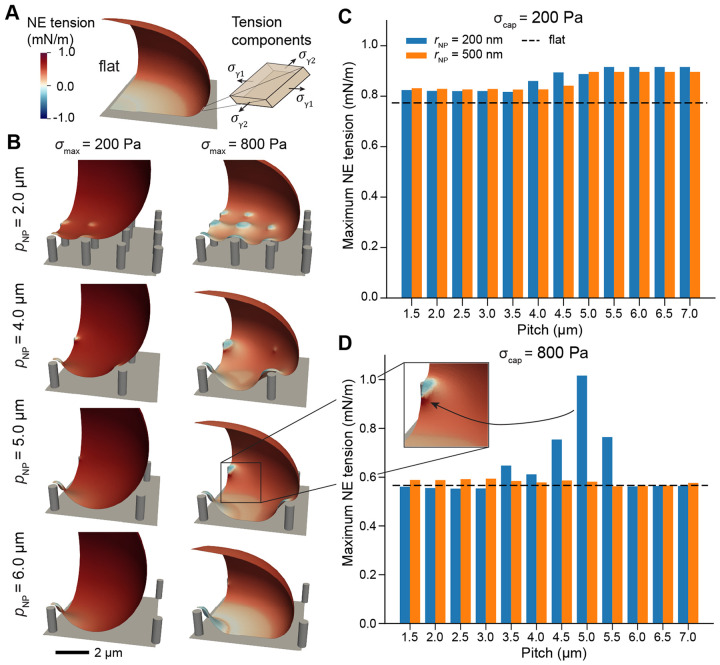
NE tension is maximized on moderate to high-pitch nanopillar substrates. A) NE tension on a flat substrate with maximum cap stress of 800 Pa. Inset schematic shows the stresses considered to approximate the tension experienced by the NE double bilayer (see Equation (13)). B) NE tension on substrates with $r_{\text{NP}}=200\;\text{nm}$, $h_{\text{NP}}=1.5\;\mu m$, and $p_{\text{NP}}=2\;\mu m$, 4μm, 5μm, or 6μm for maximum cap stress of 200 Pa or 800 Pa. C-D) Maximum NE tension as a function of nanopillar spacing for cap stresses of 200 Pa (C) or 800 Pa (D), with the dashed line indicating the maximum NE tension on a flat surface. Inset shows the region associated with maximum tension for $r_{\text{NP}}=200\;\text{nm}$, $p_{\text{NP}}=2\;\mu m$, and $\sigma_{\text{cap}}=800$ Pa. All NE tension values were computed according to Equation (13) evaluated at the midplane of the double bilayer ($\Gamma_{\text{membrane}}$ in Figure S1B).

We then determined the implication of these deformations for NE tension (Figure 3 and Movie 2). Specifically, to approximate the tension experienced by the nuclear membranes, we considered the average in-plane tension evaluated over the outer 50 nm layer of the NE shell, representing the bilayers plus the perinuclear space [52] (Figure 3A). Overall, the maximum NE tension followed a similar trend to stretch – nuclei on 200 nm radius nanopillars experiencing high cap stresses exhibited maximum tension for pitches between 5 to 6 microns (Figure 3B-D). We observed the highest tension directly adjacent to nanopillar contacts formed on these moderate pitches (Figure 3D, inset).

Altogether, this quantification was consistent with our findings for NE stretch, establishing that NE tension and strain were both maximized at moderate-to-high pitch, a consequence of the tradeoff between contacting more nanopillars at lower pitches and the increasing local stretch facilitated by wider gaps between nanopillars.

### 4.3 Nuclear compression leads to YAP/TAZ nuclear translocation

**Figure 4:**
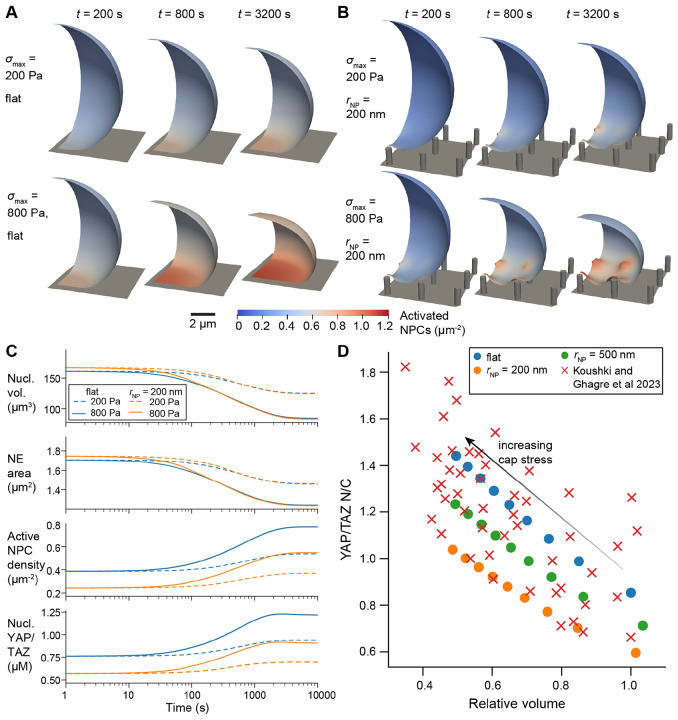
YAP/TAZ nuclear localization correlates with compression on flat or nanopillar substrates. A-B) Density of activated NPCs over time on a flat substrate (A) or on 200 nm nanopillars ($h_{\text{NP}}=1.5\;\mu m$ and $p_{\text{NP}}=3\;\mu m$, panel B) for maximum cap stresses of 200 Pa or 800 Pa. C) Plots of nuclear volume, outer NE suface area, active NPC density, and nuclear YAP/TAZ over time for each of the cases shown in panels A-B. D) Steady-state YAP/TAZ N/C versus relative nuclear volume for nuclear compression on a flat substrate or substrate with 200 nm or 500 nm radius nanopillars ($h_{\text{NP}}=1.5\;\mu m$ and $p_{\text{NP}}=3\;\mu m$), compared to data from [14]. Simulated cap stresses ranged from 0 Pa to 800 Pa (right to left). Experimental data was extracted from Fig 3C in [14], considering wild-type NIH-3T3 cells on 12 kPa polydimethylsiloxane. Experimental volumes were normalized to the maximum measured value after excluding an outlier. Simulated volumes were normalized to the nuclear volume on a flat substrate with no cap stress.

Previous experimental work suggests that NPC opening is sensitive to the stretch of the nuclear envelope [11, 12]. Accordingly, regions of high stress and strain in the NE identified above are likely sites of accelerated transport through the NPCs. To investigate this further, we coupled our mechanical model to a reaction-transport model of NPC opening, YAP/TAZ transport, and lamin localization (Figure 1E). In these simulations, we assumed that the cap stress evolved over time:

(1) $$\sigma_{\text{cap}}=\sigma_{\max}\left(1-\text{exp}\left(-\frac{t}{t_{0,\text{cap}}}\right)\right),$$

where $\sigma_{\text{max}}$ set the maximum cap stress after the cell had fully spread and $t_{0,\text{cap}}$ set the characteristic timescale of actin cap assembly. In initial tests, we fixed $t_{\text{cap}}$ to 1000 s and varied $\sigma_{\text{max}}$ from 0 Pa to 800 Pa on a flat substrate and on nanopillar substrates with $p_{\mathrm{NP}}=3\;\mu m$ and $h_{\mathrm{NP}}=1.5\;\mu m$. We found that increased cap stress led to overall reductions in the NE surface area and an associated increase in activated NPC density (Figure 4A-C, Movie 3). This aggregation occurred due to advection (Figure 1E), as well as localized activation of NPCs in regions of high F-actin around nanopillars. Enhanced NPC opening then led to a consistently higher YAP/TAZ nuclear-to-cytosolic ratio (N/C) following compression on nanopillar substrates and flat substrates alike (Figure 4C). For any given cap stress, our model predicted higher nuclear levels of YAP/TAZ in cells on flat substrates due to lower global levels of actin polymerization, in agreement with previous measurements [18, 29].

We then compared these model predictions to previous experimental measurements of nuclear volumetric compression and YAP/TAZ N/C [14]. In our simulations, we found that nuclear volume decreased concomitantly with NE surface area; accordingly, our model predicted a strong correlation between nuclear volume and NPC activation that led to YAP/TAZ nuclear localization (Figure 4C) This correlation agreed well with experimental measurements, requiring only one free parameter to scale the magnitude of the transport rate of YAP/TAZ through activated NPCs ($\phi_{\text{scale}}$ in Equation (37)). We confirmed that the correlation between compression and YAP/TAZ localization was not dependent on the degree of NPC stretch sensitivity or the relative contribution of lamin A/C to NE stiffness (Figure S4), but was mainly a geometrical effect, attributed to aggregation of species in the NE surface.

### 4.4 Nuclear loading rate and nanopillar spacing modulate laminar stress during deformation

**Figure 5:**
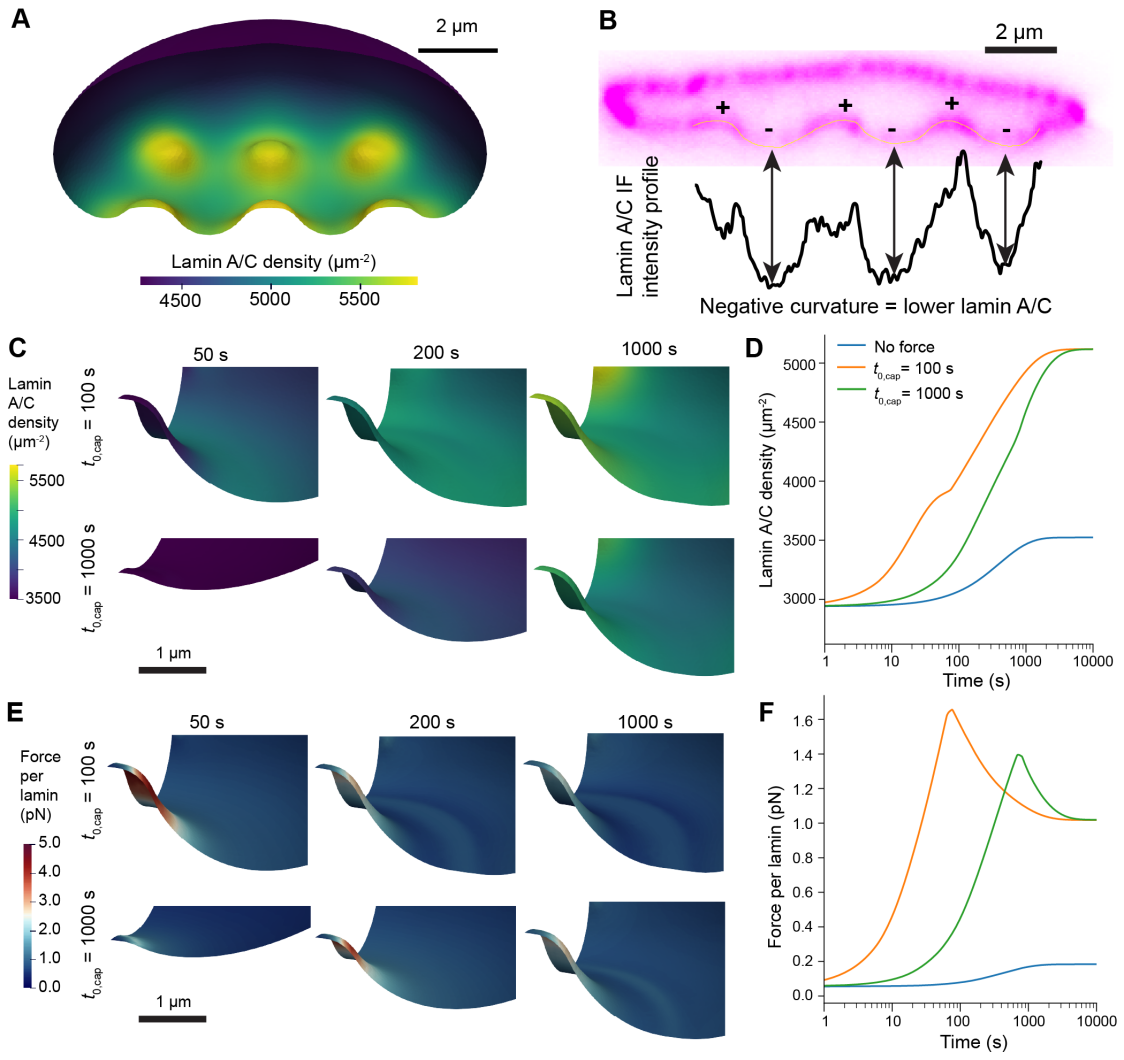
Lamin localization is modulated by the rate of actin cap assembly. A) Lamin A/C density in the NE following simulated nuclear deformation on a substrate with $r_{\text{NP}}=200\;\text{nm}$, $h_{\text{NP}}=1.5\;\mu m$ and $p_{\text{NP}}=4\;\mu m$, $\sigma_{\max}=800$ Pa. B) Side view of lamin A/C fluorescence measured via confocal microscopy in a U2OS cell (data used from [17], permission pending). Regions are annotated to denote positive (inward) and negative (outward) curvature, with the lamin A/C line intensity profile of the lower region of the NE plotted directly below. C-F) Lamin A/C density (C-D) and force per lamin (E-F) during deformation on 200 nm radius nanopillars ($h_{\text{NP}}=1.5\;\mu m$ and $p_{\text{NP}}=5\;\mu m$), $\sigma_{\max}=800$ Pa and deformation timescale $t_{0,\text{cap}}$ is 100 s or 1000 s as labeled. Visualizations in C and E show the region of the NE close to the central nanopillar at select timepoints in each simulation. Plots in D and F show the average lamin density and average force per lamin subunit over the lower region of the NE (in undeformed coordinates, X<1.5, Y<1.5, $Z<z_{\text{nuc}}+1.0$, Equation (2)).

In our biochemical model, the lamin content in the NE was tightly regulated by the rates of lamin phosphorylation and dephosphorylation and by transport due to advection and diffusion. As expected, we found that at the final time points of our simulations, lamin accumulated around nanopillars due to local actin assembly (Figure 5A), in good agreement with previous measurements of cells on nanopillar or nanoneedle substrates (Figure 5B, [17, 18, 30]). Given experimental studies establishing the importance of loading rates on lamin depletion during nuclear deformations [16, 25], we varied the characteristic timescale of deformation, $t_{0,\text{cap}}$. As outputs, we quantified the local concentration of dephosphorylated lamin in the NE and the force experienced per lamin subunit (Equation (41)). Given the established role of lamin A/C in nuclear integrity [16, 22, 50], we assumed the latter metric was a reliable indicator for the likelihood of NER.

For these tests, we considered substrates with 200 nm radius nanopillars and 5 μm pitch, as this combination led to high NE stretch and tension in the purely mechanical simulations (Figure 3D). We tested lamin remodeling for different rates of force application ($t_{0,\text{cap}}=100\;s$ or 1000 s), observing that in both cases, lamins accumulated around nanopillar-indented regions at later time points (Figure 5C-F, Movie 4). However, the rate of nuclear deformation had a strong impact on the total force experienced by the nuclear lamina over time. Naturally, the force per lamin increased more rapidly in the case of faster compression (Figure 5E-F, Movie 5). Furthermore, the peak force per lamin was also higher, as lamins were depleted at early times due to advection and less time for lamin network assembly around nanopillars (Figure 5E-F, Movies 4 and 5). This suggests that the rate of cell spreading and actin cap formation are likely to modulate the occurrence of NER.

**Figure 6:**
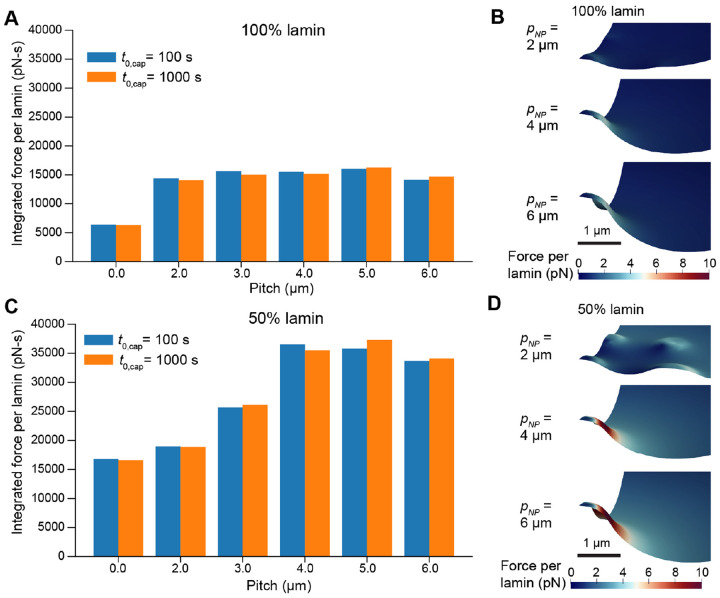
Force per lamin depends on nanopillar spacing and total lamin content. A) Integrated force (area-under-the-curve of maximum force per lamin subunit over time) as a function of nanopillar spacing. B) Snapshots of NE morphology and distribution of force per lamin during deformation on nanopillar substrates with different pitches. C-D) Integrated force and force-per-lamin visualization for cells expressing 50% less lamin than in A-B. Visualizations in B and D correspond to the time point associated with maximum force per lamin during faster deformations ($t_{0,\text{cap}}=100\;s$, $\sigma_{\text{cap}}=400$ Pa)

We proceeded to test the force per lamin over a range of different nanopillar pitches to characterize the effect of nanotopography on rupture likelihood. We integrated the maximum force per lamin over time as a metric for the cumulative likelihood of rupture (Figure 6A-B). This revealed that the forces experienced by the lamina were maximized on 4-5 μm pitch nanopillar substrates, correlating well with the outcome of the purely mechanical simulations in Figures 2 and 3. Furthermore, although the peak forces were higher for fast deformation, we found that the integrated force was comparable or even higher in the case of slow deformation (Figure 6A). This demonstrates that nanotopography is a strong predictor of NER likelihood across different nuclear loading rates.

### 4.5 Model predictions agree with measurements of nuclear rupture in low-lamin cells

Finally, we considered the potential effects of lamin knockdown on nuclear deformation and the forces experienced per lamin. We reduced the total lamin content in our simulations to 50% of the original value and simulated nuclear deformation on a range of nanopillar pitches, computing the integrated force as described above. These simulations revealed consistently higher forces experienced per lamin subunit in lamin-deficient cells, with the region of highest force still concentrated around the central nanopillar (Figure 6C-D,Movies 6 and 7). This suggests that low-lamin cells have a much higher NER likelihood compared to wild-type cells.

**Figure 7:**
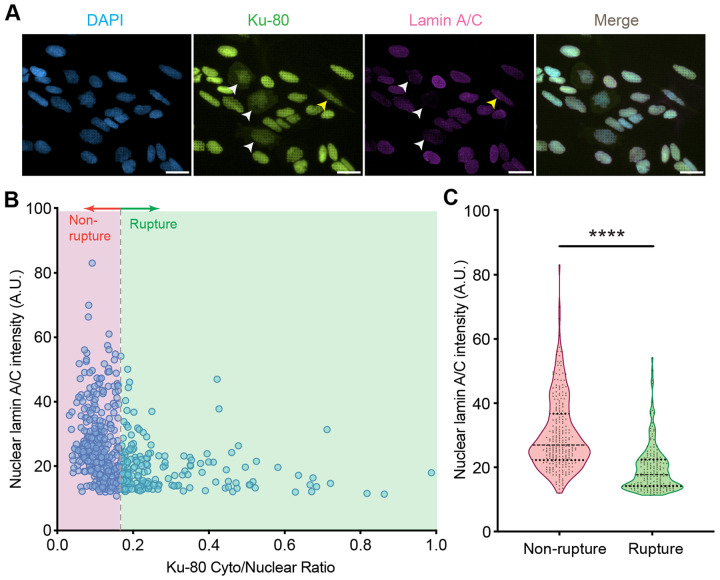
Low-lamin cells are more likely to exhibit NER on nanopillar substrates. A) Representative immunofluorescence images of U2OS cells treated with siLMNA on nanopillars. Cells were stained for DAPI (blue), Ku-80 (green), and lamin A/C (magenta). White arrows indicate cells with low lamin A/C expression that show NER evidenced by nucleus-to-cytoplasm mislocalization of Ku-80. The yellow arrow indicates a cell with normal lamin A/C expression that shows NER, consistent with stochastic rupture events on high-curvature nanotopography. Scale bar: 20 μm. B) Scatter plot characterizing the relationship between nuclear lamin A/C intensity and Ku-80 cytoplasmic-to-nuclear ratio (C/N) across the heterogeneous cell population. The vertical dashed line indicates the Ku-80 ratio threshold used to classify ruptured versus non-ruptured cells. C) Quantification of lamin A/C intensity for ruptured versus non-ruptured subpopulations classified in (B). **** denotes p ≤ 0.0001 according to a Student’s *t*-test and dashed horizontal lines in panel C indicate medians and upper and lower quartiles.

To validate these modeling predictions, we cultured U2OS cells on engineered quartz nanopillar substrates (height 3.24 ± 0.25 μm, diameter 0.90 ± 0.016 μm, and pitch 3.49 ± 0.06 μm). We reduced lamin A/C expression using small interfering RNA (siRNA) targeting the LMNA gene. The treated population showed heterogeneous lamin A/C expression, with many cells exhibiting a significant reduction in lamin content compared to controls (Figure 7A). To examine nuclear mechanical integrity across a wide range of lamin expression levels, we pooled single-cell data from both the siLMNA treated and non-silenced control populations (Figure 7B). Cells were immunostained for Ku-80 to detect NER via protein mislocalization from the nucleus to the cytoplasm. Using a data-driven Gaussian Mixture Model (GMM) on this combined population, we determined a Ku-80 cytoplasmic-to-nuclear ratio (C/N) of 0.16 as the threshold distinguishing ruptured from non-ruptured nuclei. This analysis revealed that low-lamin cells were much more abundant in the population with ruptured nuclei, suggesting that reduced lamin content increases rupture likelihood. This finding agrees with our simulations, confirming that low lamin A/C density compromises nuclear integrity.

## 5 Discussion

Cell motility in physiological environments is strongly influenced by nuclear size and deformability. In contrast to many classic motility assays, in which cells freely migrate over flat 2D surfaces, migration through pericellular spaces in vivo requires cells to squeeze through small passages and adapt to nanoscale curvature [53-55]. In addition to the well-appreciated role of PM curvature in modulating protein aggregation and cellular signaling [53, 56], recent studies indicate that changes in nuclear curvature influence biochemical events at the NE [16, 57]. For instance, rapid induction of Gaussian curvature leads to dilution of lamin A/C and lamin B within the NE, increasing the likelihood of rupture [16]. Independent of curvature, nuclear compression leads to nuclear volume loss [14, 33, 58] and increased likelihood of rupture [15]. Our study integrates these recent discoveries within a single modeling framework, elucidating the effects of compression and imposed curvature on stresses experienced by the nucleus and on remodeling of the nuclear lamina.

Our model of the NE relies on a hyperelastic formulation that supports large deformations, as utilized in several previous models [7, 33, 34, 37]. This approach incorporates more detailed mechanics of the nuclear surface than other recent models of the nucleus as a droplet with surface tension [13]. Our mechanical model alone does not explicitly include viscous terms, but we consider time-dependent changes in NE stiffness due to lamin reaction and transport, which effectively captures some degree of viscous remodeling. A major innovation in our approach lies in the coupling between nuclear mechanics and biochemical reaction-transport. Using our recently developed software framework [42] and state-of-the-art finite element analysis in FEniCS [59], we were able to recapitulate several key features of nuclear mechanotransduction in silico.

Our simulations make clear predictions about changes in YAP/TAZ nucleocytoplasmic transport, a well-established player in mechanotransduction signaling [60, 61]. In particular, we show that nuclear compression results in volume loss and overall contraction of the NE, resulting in higher levels of YAP/TAZ in the nucleus. Surprisingly, this effect did not require explicit addition of stretch-sensitivity for NPCs in our model, but was mainly attributed to the aggregation of lamin A/C and activated NPCs following NE contraction. This suggests that enhanced opening of NPCs can be achieved by strengthening nucleoskeleton-cytoskeleton linkages and may not require external stretch. Indeed, enhanced transport through NPCs was shown to partially rely on transmission of forces from the cytoskeleton to the NE via the Linker of the Nucleoskeleton and Cytoskeleton (LINC) complex [11-13]. The overall relationship between NE deformation and NPC opening likely involves a complex interplay between NE curvature, NE stretch, and nucleoskeleton-cytoskeleton interactions. The predicted effects of nanotopography on nucleocytoplasmic transport in this work may also extend to other known mechanosensitive transcription regulatory factors such as TWIST1 [12, 62].

Our current model considers many key features of nuclear mechanics established in the literature; for instance, lamin redistribution was assumed to change the local NE stiffness [23] and also determined the force experienced by the NE, dictating the likelihood of NER [13, 16, 25]. However, many other molecular constituents not explicitly considered here also play important roles in nuclear mechanics. Although the LINC complex is implicitly represented by interactions between actin and lamin in our model (Figure 1E), we do not evaluate the direct contribution of LINC-mediated connections to the NE tension. This may help explain why the tension approximated by our model (Figure 3, up to 1 mN/m) is higher than previously estimated [63, 64]. Furthermore, our current estimates of tension neglect the detailed wrinkling of the NE observed in some experiments and models [13, 33, 40]. The surface area of the outer NE in our model reflects the apparent surface area, whereas the total membrane surface area is likely much larger, suggesting that our calculation of the tension may be an overestimate. Finally, our model does not consider the role of chromatin reorganization in nuclear mechanics. Motors such as BRG1 have recently been shown to strongly alter nuclear stiffness [65, 66], but further data is required to constrain models that include such proteins in descriptions of nuclear mechanics.

Our model predicts that an intermediate nanopillar pitch (4–5 μm) maximizes NE stress and stretch. These mechanically stressed states are expected to increase transient nuclear permeability through stretch-mediated NPC opening and, in some cases, NE rupture. This provides a geometry-guided design parameter for nuclear delivery and can facilitate nuclear access to cargoes that are otherwise transport-limited, such as large protein complexes (CRISPR RNPs), donor DNA templates, and transcriptional modulators (e.g., TEAD inhibitors) [67]. More broadly, these predictions reinforce the importance of nanotopography in shaping cellular responses in fibrous matrices where cells experience curvature-driven stress focusing at ECM contact points.

Our work has natural implications for the class of diseases known as laminopathies, which are associated with mutations in lamin A/C [26, 68]. Most laminopathies are associated with changes in nuclear morphology [24, 68, 69], suggesting changes in mechanical properties of the nuclear lamina. This may be attributed to changes in protein structure or in lamin phosphorylation state, leading to altered abundance of lamins in the NE [25, 70]. As shown by our simulations with low-lamin cells and experiments with lamin-depleted cells, reduced lamin content can render cells more susceptible to NER. In fact, several studies have measured increased prevalence of NER in lamin mutant cells [71-73]. Outside of the context of disease, lamin expression levels vary considerably across different tissues [25]. Our modeling framework could therefore be used to help elucidate changes in nuclear mechanics and mechanotransduction observed in cell types across different organs and tissues in the human body.

## 6 Methods

### 6.1 Model Formulation

#### 6.1.1 Hyperelastic model of the nuclear envelope

In line with previous studies, we model the NE as a hyperelastic shell with finite thickness [7, 33, 34]. This approach lumps the double bilayer with the underlying nuclear lamina consisting largely of a network of lamin A/C. The stress-free configuration is a spherical shell with center at $\left[0,0,z_{\mathrm{nuc}}+R_{0}\right]$, outer radius $R_{0}$, and thickness ΔT:

(2) $$X^{2}+Y^{2}+{\left(Z-z_{\text{nuc}}-R_{0}\right)}^{2}=R_{0}^{2}\;\text{on}\;\Gamma_{\text{outer}},$$

(3) $$X^{2}+Y^{2}+{\left(Z-z_{\text{nuc}}-R_{0}\right)}^{2}={\left(R_{0}-\Delta T\right)}^{2}\;\text{on}\;\Gamma_{\text{inner}},$$

where X=[X,Y,Z] are the undeformed material coordinates (Figure S1A). The volume between $\Gamma_{\text{inner}}$ and $\Gamma_{\text{outer}}$ is $\Omega_{\mathrm{NE}}$ (Figure S1B). N, the unit normal of the reference geometry (Figure S1A), can be expressed analytically over any slice of the NE with constant radius:

(4) $$N=\frac{1}{\sqrt{X^{2}+Y^{2}+{\left(Z-z_{\text{nuc}}-R_{0}\right)}^{2}}}[X,Y,Z-z_{\text{nuc}}-R_{0}].$$

Given a displacement field u defined within $\Omega_{\mathrm{NE}}$ resulting in new deformed coordinates x=X+u, we define conventional displacement metrics including the deformation gradient tensor:

(5) $$F={\left(\frac{\partial x}{\partial X}\right)}^{T},$$

and the right Cauchy-Green tensor:

(6) $$C=F^{T}\cdot F.$$

These tensors are associated with the following strain invariants:

(7) $$I_{1}=\text{tr}\;C,$$

(8) $$I_{2}=\frac{1}{2}\;[{\left(\text{tr}\;C\right)}^{2}-\text{tr}(C\cdot C)]\;,$$

(9) J=detF,

where the final invariant is the Jacobian of transformation representing volume dilatation. The strain energy functional is given in terms of these invariants assuming an incompressible Mooney-Rivlin material as in [34]:

(10) $$W=E_{1}(I_{1}-3)+E_{2}(I_{2}-3)-p(J-1),$$

where $E_{1}$ and $E_{2}$ are elastic constants (values for these and other parameters given in Table 1) and p is a pressure-like variable that acts as a Lagrange multiplier to enforce incompressibility within the shell. The first Piola-Kirchoff stress tensor (t) and the Cauchy stress tensor (σ) can then be computed by:

(11) $$t=\frac{\partial W}{\partial F^{T}},$$

(12) $$\sigma =\frac{1}{J}F\cdot \frac{\partial W}{\partial F^{T}}.$$

We also consider the surface tension at the outer edge of the NE as a scalar output for the Cauchy stress tensor:

(13) $$\gamma =\frac{\Delta \hat{z}}{2}(\sigma_{\gamma 1}+\sigma_{\gamma 2})$$

(14) $$\text{where}\;\sigma_{\gamma 1}=\frac{(e_{\Theta }\cdot t)\cdot e_{\Theta }}{\alpha_{\Theta }}$$

(15) $$\text{and}\;\sigma_{\gamma 2}=\frac{(e_{\Phi }\cdot t)\cdot e_{\Phi }}{\alpha_{\Phi }}.$$

The unit vectors in the Θ and Φ directions (Figure S1A), as well as the stretch in the associated planes are given by:

(16) $$e_{\Theta }=\frac{1}{\sqrt{X^{2}+Y^{2}}}[-Y,X,0],$$

(17) $$e_{\Phi }=e_{\Theta }\times N,$$

(18) $$\alpha_{\Theta }=|Je_{\Phi }\cdot F^{-1}|,$$

(19) $$\alpha_{\Phi }=|Je_{\Theta }\cdot F^{-1}|.$$

Assuming mechanical equilibrium and no body forces, the following equation must be satisfied:

(20) $$\nabla \cdot t=0\;\;\text{in}\;\Omega_{\text{NE}},$$

We assume the center of the nucleus is positioned directly above a central nanopillar, leading to a symmetry condition about the x and y axes:

(21) $$u_{y}=0\;\;\text{for}\;x=0,$$

(22) $$u_{x}=0\;\;\text{for}\;y=0,$$

such that we only need to simulate a quarter of the nuclear geometry. Any region of the NE that contacts the lower region of the substrate experiences an additional Dirichlet condition:

(23) $$u_{z}=-h_{\text{NP}}-Z\;\;\text{for}\;z\leq -h_{\text{NP}},$$

where coordinates are defined such that the bottom of the NE rests at $z=-h_{\mathrm{NP}}$.

To model nanopillar-mediated indentation, we apply a repulsive potential acting between the PM adhered to the nanopillar surface and the outer NE:

(24) σcontact={σcontact,0exp(−Z+uz−dstericdsteric)onΓcontact0otherwise},

where $\Gamma_{\text{contact}}$ is the portion of the outer NE directly above a nanopillar contact (Figure S1B, Equation (44)) and $d_{\text{steric}}$ is the characteristic distance associated with steric repulsion. This provides a Robin boundary condition for the z-displacement, which is supplemented by an additional Dirichlet boundary condition for the z-displacement at the cap that was found to aid in numerical stability (Equation (47)).

The above Robin boundary condition is grouped with other stress-type boundary conditions including stress on the outer NE $\left(\Gamma_{\text{outer}}\right)$ due to the perinuclear actin cap and stress at the interior nuclear surface $\left(\Gamma_{\text{inner}}\right)$ due to an osmotic pressure gradient. To apply these conditions, we note that the surface traction at the inner and outer boundaries of the NE is given by:

(25) $$\sigma_{\text{NE}}=\frac{N\cdot t}{\alpha },$$

where α is the local stretch ratio of the nuclear surface, needed to convert the psuedotraction stress to a true traction over the deformed surface [45]. Accordingly:

(26) N⋅t={−ασcap,totezonΓouter∖ΓcontactαΔPnonΓinnerα(σcontact−σcap,tot)ezonΓcontact},

where $e_{z}$ is the unit vector in the z direction and n is the unit normal associated with the deformed surface (Figure S1B). α and n are computed using Nanson’s formula, which relates the area and unit normal (N, Equation (4)) in the reference configuration to those in the deformed configuration:

(27) $$\alpha =|JN\cdot F^{-1}|,$$

(28) $$n=\frac{JN\cdot F^{-1}}{\alpha }.$$

$\sigma_{\text{cap}}$ varies over the geometry, reaching its maximum value at the top of the nucleus and always directed in the z-direction:

(29) $$\sigma_{\text{cap},\text{tot}}=\sigma_{\text{cap}}\text{exp}\left(\frac{Z-(z_{\text{nuc}}+2R_{0})}{Z_{0}}\right),$$

where $\sigma_{\text{cap}}$ sets the force magnitude and $Z_{0}$ determines the spatial extent of the cap stress.

When solving the system, we first introducing an osmotic pressure gradient and then increasing $\sigma_{\text{cap}}$ in small increments. As cap stress increases, pressure evolves over each iteration to penalize changes in nuclear volume:

(30) $$\Delta P=\Delta P_{\text{prev}}-\phi_{\text{inner}}\frac{{\text{vol}}_{\text{nuc}}-{\text{vol}}_{\text{nuc},\text{prev}}}{{\text{vol}}_{\text{nuc}}},$$

where $\phi_{\text{inner}}$ is the bulk modulus of the nucleoplasm, which was set to ensure maximum volume loss of about 50% in line with previous measurements [14, 33]. $\Delta P_{\mathrm{prev}}$ is the pressure gradient at the previous iteration and ${\mathrm{vol}}_{\mathrm{nuc}}$ and ${\mathrm{vol}}_{\mathrm{nuc},\mathrm{prev}}$ are the nucleoplasmic volumes at the current iteration and the previous iteration, respectively.

#### 6.1.2 Biochemical reaction and transport

We consider the reaction-transport of several molecular species with the NE (lamin A/C and NPCs) or within the nucleoplasm (YAP/TAZ and phosphorylated lamin A/C). Dephosphorylated lamin A/C is defined on the inner surface $\left(\Gamma_{\text{inner}}\right)$, whereas NPC species are defined on the outer NE surface $\left(\Gamma_{\text{outer}}\right)$. However, because we assume that all interactions between lamins, NPCs, YAP/TAZ, actin, and myosin occur at the outer surface of the NE, we track the projected lamin concentration on $\Gamma_{\text{outer}}$ as detailed below. We do not explicitly include the cytosolic space in our model, but we do consider the cytosolic concentrations of F-actin ([F]), activated myosin (${\left[M\right]}_{A})$, and free cytosolic YAP/TAZ $\left({\left[Y\right]}_{\text{free}}\right)$ at the NE interface with the cytosol. We assume cytosolic YAP/TAZ and activated myosin are well-mixed, as YAP/TAZ diffuses rapidly in the cytosol [76, 77] and myosin activation is determined by Rho-associated protein kinase in our previous models, which also diffuses rapidly in the cytosol [78]. Myosin is fixed according to values from our previous simulations [18], and cytosolic YAP/TAZ species are computed at each time point via Equations (83) and (84). F-actin concentration is expressed as a function of distance between the NE and the PM $\left(d_{\mathrm{NE}\text{-}\mathrm{PM}}\right)$ and of curvature of the closest point on the PM $\left(H_{\mathrm{PM}}\right)$ to capture the trends modeled in our previous work [18]:

(31) $$[F]={\left[F\right]}_{0}+{\left[F\right]}_{1}\;\text{exp}\left(-\frac{d_{\text{NE-PM}}}{d_{F}}\right)\text{exp}\left(\frac{H_{\text{PM}}}{H_{0}}\right).$$

${\left[F\right]}_{0}$, ${\left[F\right]}_{1}$, $d_{F}$, and $H_{0}$ were constrained according to our previous model; values and definitions for these parameters and all others are provided in Table 2. Global F-actin concentration, ${\left[F\right]}_{0}$, varies according to the nanopillar radius to capture the curvature-dependent inhibition of focal adhesion formation shown in previous experiments [29, 79] and included in our previous model [18]. The PM-NE distance and curvature of the PM were computed assuming an ideal cylindrical geometry of the PM around the nanopillar and only considering the closest nanopillar to a given point on the NE as fully detailed in Equation (87).

Given these cytosolic variables, the reaction-transport equation governing dephosphorylated lamin in the NE ([L]) is:

(32) $$\frac{\partial [L]}{\partial t}+\nabla_{S,\text{inner}}\cdot (v_{\text{NE},\text{inner}}[L])=D_{L}\nabla_{S,\text{inner}}^{2}[L]-k_{\text{rl}}[L]+k_{\text{fl}}\frac{p_{\text{cyto}}{\left[F\right]}^{2.6}}{C_{L}+p_{\text{cyto}}{\left[F\right]}^{2.6}}{\left[L\right]}_{\text{phos}}\;\;\text{on}\;\;\Gamma_{\text{outer}}.$$

Note that while the surface differential operator $\left(\nabla_{S,\text{inner}}\right)$ and the advection velocity $\left(v_{\mathrm{NE},\text{inner}}\right)$ are defined according to the motion of the inner surface $\Gamma_{\text{inner}}$, the concentration itself is computed on the outer surface $\Gamma_{\text{outer}}$, allowing all reactions to occur locally over this interface. $\nabla_{S,\text{inner}}$ and $v_{\mathrm{NE},\text{inner}}$ are defined in Section 6.2.3. Lamin diffuses with coefficient $D_{L}$ and phosphorylation and dephosphorylation are described using relations from our previous work [80] (see definitions and values of $k_{\mathrm{rl}}$, $k_{\mathrm{fl}}$, $p_{\mathrm{cyto}}$, and $C_{L}$ in Table 2). In contrast to our previous model, we consider phosphorylated lamin concentration $\left({\left[L\right]}_{\text{phos}}\right)$ within the nuclear volume, as it is assumed disassembled and effectively solubilized [21, 50]:

(33) $$\frac{\partial {\left[L\right]}_{\text{phos}}}{\partial t}+\nabla \cdot (v_{\text{nuc}}{\left[L\right]}_{\text{phos}})=D_{L,\text{vol}}\nabla^{2}{\left[L\right]}_{\text{phos}}\;\;\text{in}\;\;\Omega_{\text{full}},$$

(34) $$D_{L,\text{vol}}\nabla {\left[L\right]}_{\text{phos}}\cdot n=\frac{1}{N_{\text{conv}}}\left(k_{\text{rl}}[L]-k_{\text{fl}}\frac{p_{\text{cyto}}{\left[F\right]}^{2.6}}{C_{L}+p_{\text{cyto}}{\left[F\right]}^{2.6}}{\left[L\right]}_{\text{phos}}\right)\;\;\text{on}\;\;\Gamma_{\text{outer}},$$

where $v_{\mathrm{nuc}}$ is the extrapolated velocity field within the nucleus (see Section 6.2) and $D_{L,\mathrm{vol}}$ is the diffusion coefficient of phosphorylated lamin A/C in the nucleoplasm. $\Omega_{\mathrm{full}}$ includes both volumetric domains $\left(\Omega_{\mathrm{full}}=\Omega_{\mathrm{nuc}}\cup \Omega_{\mathrm{NE}}\right)$, which are combined for the case of volume species in the biochemical model.

Activated $\left({\left[NPC\right]}_{A}\right)$ and deactivated ([NPC]) NPCs are also included in our framework, advected with the outer surface of the NE (velocity $v_{\mathrm{NE},\text{outer}}$):

(35) $$\frac{\partial [NPC]}{\partial t}+\nabla_{S}\cdot (v_{\text{NE},\text{outer}}[NPC])=D_{NPC}\nabla_{S}^{2}[NPC]-k_{\text{fNPC}}[F][L]{\left[M\right]}_{A}[NPC]+k_{\text{rNPC}}{\left[NPC\right]}_{A}\;\text{on}\;\Gamma_{\text{outer}},$$

(36) $$\frac{\partial {\left[NPC\right]}_{A}}{\partial t}+\nabla_{S}\cdot (v_{\text{NE},\text{outer}}{\left[NPC\right]}_{A})=D_{NPC}\nabla_{S}^{2}{\left[NPC\right]}_{A}+k_{\text{fNPC}}[F][L]{\left[M\right]}_{A}[NPC]-k_{\text{rNPC}}{\left[NPC\right]}_{A}\;\text{on}\;\Gamma_{\text{outer}},$$

where $D_{\mathrm{NPC}}$ is the diffusion coefficient of NPCs and $k_{\mathrm{fNPC}}$ and krNPC are the rates of NPC activation and deactivation, respectively. $\nabla_{S}$ is the surface gradient operator as defined in Equation (55).

The resulting transport of YAP/TAZ across the NE depends on nuclear stretch, such that the maximum rate of transport is a function of outer NE stretch α (Equation (27)):

(37) $$k_{\text{in},\text{tot}}=\phi_{\text{scale}}(k_{\text{in},b}+k_{\text{in}}{\left[NPC\right]}_{A})\;\text{exp}\left(\frac{\alpha -\alpha_{\text{init}}}{\alpha_{0}}\right),$$

where $\alpha_{\mathrm{init}}$ is the average NE stretch at t=0 (after increasing the nuclear pressure), $k_{\mathrm{in},b}$ and $k_{\mathrm{in}}$ are the baseline and NPC-activation dependent import rates, and $\alpha_{0}$ determines the stretch sensitivity. $\phi_{\text{scale}}$ is a flux scaling factor included as a calibration parameter for this revised model. This results in the following equation and boundary condition for nuclear YAP/TAZ $\left({\left[Y\right]}_{\text{nuc}}\right)$:

(38) $$\frac{\partial {\left[Y\right]}_{\text{nuc}}}{\partial t}+\nabla \cdot (v_{\text{nuc}}{\left[Y\right]}_{\text{nuc}})=D_{Y}\nabla^{2}{\left[Y\right]}_{\text{nuc}}\;\;\text{in}\;\Omega_{\text{nuc},\text{tot}},$$

(39) $$D_{Y}\nabla {\left[Y\right]}_{\text{nuc}}\cdot n=\frac{1}{N_{\text{conv}}}(k_{\text{in},\text{tot}}{\left[Y\right]}_{\text{free}}-k_{\text{out}}{\left[Y\right]}_{\text{nuc}})\;\;\text{on}\;\Gamma_{\text{outer}},$$

where $D_{Y}$ is the nuclear diffusion coefficient of YAP/TAZ and $k_{\text{out}}$ is the rate of YAP/TAZ efflux from the nucleus.

#### 6.1.3 Mechanochemical coupling

We introduced time-dependence into our mechanical system by writing the cap stress as a function of time (Equation (1)). Solving for associated nuclear deformations at over a given time step then determines the velocities $v_{\mathrm{NE},\text{outer}}$, $v_{\mathrm{NE},\text{inner}}$, $v_{\mathrm{nuc}}$ (via extrapolation as described in Section 6.2.2) and the NE stretch α, all of which appear in the above biochemical reaction-transport equations. We also consider coupling from the biochemical model to the mechanical model via lamin-dependent changes in NE stiffness. Based on measured correlations between lamin A/C abundance and nuclear bulk modulus [23], we scale both elastic moduli ($E_{1}$ and $E_{2}$ in Equation (10)) by factor $E_{\text{scale}}$:

(40) $$E_{\text{scale}}=(\phi_{L}-1)+\phi_{L}\frac{\left[L\right]}{{\left[L\right]}_{\text{ref}}},$$

where $\phi_{L}$ is a fraction between 0 and 1 that determines the relative contribution of lamin A/C to NE stiffness.

As a main output relevant to mechanochemical coupling in Figures 5 and 6, we compute the force per lamin subunit, given by normalizing local stress to lamin density:

(41) $$F_{L}=\frac{\sigma_{\text{NE}}}{\left[L\right]}.$$

### 6.2 Numerical Methods

#### 6.2.1 Numerical approximation of hyperelastic nuclear deformation

To solve the hyperelastic equations via FEniCS, we recast them into their associated weak form:

(42) $$\int \nabla v\cdot td\Omega +\int q\;(\phi_{0}(J-1)+p)\;d\Omega +\int_{\Gamma_{\text{outer}}}\alpha \sigma_{\text{cap},\text{tot}}v\cdot e_{z}d\Gamma \;\;-\int_{\Gamma_{\text{contact}}}\alpha \sigma_{\text{contact}}v\cdot e_{z}d\Gamma -\int_{\Gamma_{\text{inner}}}J\Delta Pv\cdot (N\cdot F^{-1})d\Gamma =0,$$

where u, v are trial and test functions for displacement and p, q are trial and test functions for pressure. pressure is only specified up to an additive constant, and its boundary condition is given as:

(43) $$n\cdot \nabla p=0\;\;\text{on}\;\;\Gamma_{\text{inner}}\;\text{and}\;\Gamma_{\text{outer}}.$$

This mixed pressure-displacement form is consistent with a compressible formulation in which the bulk modulus is $\phi_{0}$. We choose $\phi_{0}$ to be sufficiently large to approximate incompressibility with sufficient accuracy (less than 0.01% deviation in NE volume). With the correct choice of function spaces (linear for pressure, quadratic for displacements), this approach mitigates numerical instabilities when solving incompressible problems via the finite element method [86, 87].

The domain $\Gamma_{\text{contact}}$ evolves over time and/or iteration number during simulations, according to the proximity of a material point to nanopillars. For implementation, it is advantageous to rewrite integrals of any function over this region (ℱ) to avoid discrete updates to the domain at each step:

(44) ∫ΓcontactℱdΓ=∫ΓouterℱℋNP(x,y)dΓ,whereℋNP(x,y)=∑nx=−NNPNNP(∑ny=−NNPNNP{1if(x−nxpNP)2+(y−nypNP)2≤rNP0otherwise}),

where $p_{\mathrm{NP}}$ and $r_{\mathrm{NP}}$ are the pitch and radius of the nanopillars on a given substrate and $N_{\mathrm{NP}}=\text{round}\left(\frac{r_{\text{max}}}{p_{\mathrm{NP}}}\right)$ for $r_{\text{max}}$ setting a maximum length cutoff. Provided the nanopillars do not overlap $\left(p_{\mathrm{NP}}\geq 2r_{\mathrm{NP}}\right)$, ${ℋ}_{\mathrm{NP}}(x,y)$ is equal to one for a region of the NE directly above a nanopillar and is zero otherwise. To avoid sharp cutoffs in our simulation, we define a smooth approximation of ${ℋ}_{\mathrm{NP}}$:

(45) $${ℋ}_{\text{NP},\text{smooth}}(x,y)=\sum_{n_{x}=-N_{\text{NP}}}^{N_{\text{NP}}}\left(\sum_{n_{y}=-N_{\text{NP}}}^{N_{\text{NP}}}\text{exp}\left(-\frac{{\left({\left(x-n_{x}p_{\text{NP}}\right)}^{2}+{\left(y-n_{y}p_{\text{NP}}\right)}^{2}\right)}^{2}}{{\left(r_{\text{NP}}+0.05\right)}^{4}}\right)\right),$$

where super-Gaussian functions are used to approximate the nanopillar domain locations (Figure S1D). Therefore, the fourth term in the above weak form is rewritten as:

(46) $$\int_{\Gamma_{\text{contact}}}J\sigma_{\text{contact}}[F]{\left[M\right]}_{\text{act}}v\cdot (N\cdot F^{-1})d\Gamma \approx \int_{\Gamma_{\text{outer}}}{ℋ}_{\text{NP},\text{smooth}}(x,y)J\sigma_{\text{contact}}[F]{\left[M\right]}_{\text{act}}v\cdot (N\cdot F^{-1})d\Gamma .$$

We found that this Robin condition on z-displacement was not sufficient to ensure convergence. Therefore, we supplemented the condition with an additional Dirichlet condition over the uppermost region of the NE at each time step:

(47) $$u_{z}=\frac{\int_{\Gamma_{\text{upper},2}}\alpha u_{z,\text{prev}}d\Gamma }{\int_{\Gamma_{\text{upper},2}}\alpha d\Gamma }\;\;\text{on}\;\;\Gamma_{\text{upper},1},$$

where $\Gamma_{\text{upper},1}$ contains the surface elements of the outer NE directly connected to the uppermost point of the reference geometry and:

(48) $$\Gamma_{\text{upper},2}=\Gamma_{\text{upper}}\setminus \Gamma_{\text{upper},1},$$

for $\Gamma_{\text{upper}}$ the region of the upper NE over which $X^{2}+Y^{2}\leq D_{\text{roof}}^{2}$ (Figure S1C). $u_{z,\mathrm{prev}}$ is the z displacement at the previous time point.

#### 6.2.2 Numerical approximation of reaction-transport

To readily facilitate mechanochemical coupling in our equations, we express all of the reaction-transport equations in terms of Lagrangian (material) coordinates. The deformation field u at the NE is given by the hyperelastic equations above. We extend the displacment field to the nucleoplasm by solving for $u_{\mathrm{nuc}}$, the displacement associated with a neo-Hookean material within the nucleus. Keeping notation consistent with the equations above, the nucleoplasmic domain $\Omega_{\mathrm{nuc}}$ is the region in which $X^{2}+Y^{2}+(Z-z_{\mathrm{nuc}}-R_{0})\leq R_{0}-\Delta T$ (Figure S1B). Defining deformation gradient tensor $F_{\mathrm{nuc}}=\left(\frac{\partial }{\partial X}(X+u_{\mathrm{nuc}})\right)$, right Cauchy-Green tensor $C_{\mathrm{nuc}}=F_{\mathrm{nuc}}^{T}\cdot F_{\mathrm{nuc}}$, and their invariants $\left(I_{1,\mathrm{nuc}}=\mathrm{tr}\;C_{\mathrm{nuc}},J_{\mathrm{nuc}}=\det\;F_{\mathrm{nuc}}\right)$, the associated strain energy function is:

(49) $$W_{\text{nuc}}=E_{1,\text{nuc}}(I_{1,\text{nuc}}-3)+\phi_{\text{nuc}}{\left(J_{\text{nuc}}-1\right)}^{2},$$

where $E_{1,\mathrm{nuc}}$ is the shear modulus and $\phi_{\mathrm{nuc}}$ is the bulk modulus, both chosen according to initial numerical testing (Table 1). Dirichlet boundary conditions are specified by the deformation field at the NE:

(50) $$u_{\text{nuc}}=u\;\;\text{on}\;\;\Gamma_{\text{inner}}.$$

The associated weak form is:

(51) $$\int \nabla v_{\text{nuc}}\cdot \frac{\partial W_{\text{nuc}}}{\partial F^{T}}d\Omega =0,$$

where $v_{\mathrm{nuc}}$ is a test function. As such, we have a global Lagrangian mapping from the reference mesh to the deformed geometry:

(52) $$dx=dX\cdot F^{T},$$

where F is now defined to extend over the entire nuclear volume; that is:

(53) F={(∂∂X(X+u))TinΩNE(∂∂X(X+unuc))TinΩnuc}.

We define differential operators in terms of this transformation. Writing $\nabla ≔\frac{\partial }{\partial x}$ and $\bar{\nabla }≔\frac{\partial }{\partial X}$:

(54) $$\nabla =F^{-T}\cdot \bar{\nabla }.$$

For surface gradients, we must account for changes in the direction of the normal associated with deformations, leading to the relation:

(55) $$\nabla_{S}=\nabla -n(n\cdot \nabla )=F^{-T}\cdot \bar{\nabla }-{\left(\frac{J}{\alpha }\right)}^{2}(N\cdot F^{-1})\left(\left(N\cdot F^{-1})\cdot (F^{-T}\cdot \bar{\nabla }\right)\right).$$

Similarly, we can define integrals in terms of Lagrangian coordinates. In particular, the $L^{2}(O)$-inner product over physical space (given as an integral over the reference domain $\Omega_{\mathrm{full}}$) is:

(56) $${\langle a,b\rangle }_{\Omega_{\text{full}}}=\int_{\Omega_{\text{full}}}a\cdot b\;Jd\Omega .$$

The $L^{2}(O)$-inner product written as an integral over reference surface $\Gamma_{\text{outer}}$ is:

(57) $${\langle a,b\rangle }_{\Gamma_{\text{outer}}}=\int_{\Gamma_{\text{outer}}}a\cdot b\alpha d\Gamma .$$

We then consider the weak form for a volume species within the nucleoplasm u (either nuclear YAP/TAZ or phosphorylated lamin here):

(58) $${\langle \partial_{t}u,\phi \rangle }_{\Omega_{\text{nuc}}}+{\langle D_{u}\nabla u,\nabla \phi \rangle }_{\Omega_{\text{nuc}}}+{\langle uv_{\text{nuc}},\nabla \phi \rangle }_{\Omega_{\text{nuc}}}-{\langle f_{u},\phi \rangle }_{\Omega_{\text{nuc}}}-{\langle R_{u},\phi \rangle }_{\Gamma_{\text{outer}}}=0,$$

and for a surface species in the NE, v (activated or inactivated NPCs here):

(59) $${\langle \partial_{t}v,\psi \rangle }_{\Gamma_{\text{outer}}}+{\langle D_{v}\nabla_{S}v,\nabla_{S}\psi \rangle }_{\Gamma_{\text{outer}}}+{\langle vv_{\text{NE},\text{outer}},\nabla_{S}\psi \rangle }_{\Gamma_{\text{outer}}}-{\langle g_{v},\psi \rangle }_{\Gamma_{\text{outer}}}=0,$$

where ϕ and ψ are test functions associated with u and v, respectively, $f_{u}$ is the volumetric reaction rate of y, $R_{u}$ is the surface reaction rate for u, and $g_{v}$ is the surface reaction rate for v. These are expressed in a fully Lagrangian fashion by using the above definitions of the inner products (Equations (56) and (57)) and differential operators (Equations (54) and (55)).

In practice, we solve each system in two steps, first solving the implicit Euler time-discretized version of the reaction-diffusion system over the fixed domain at time t:

(60) $$\frac{1}{\Delta t}{\langle u_{D}(t)-u(t-\Delta t),\phi \rangle }_{\Omega_{\text{nuc}}}+{\langle D_{u}\cdot \nabla u_{D}(t),\nabla \phi \rangle }_{\Omega_{\text{nuc}}}-{\langle f_{u}(t),\phi \rangle }_{\Omega_{\text{nuc}}}-{\langle R_{u}(t),\phi \rangle }_{\Gamma_{\text{NE}}}=0,$$

(61) $$\frac{1}{\Delta t}{\langle v_{D}(t)-v(t-\Delta t),\psi \rangle }_{\Gamma_{\text{outer}}}+{\langle D_{v}\nabla_{S}v_{D}(t),\nabla_{S}\psi \rangle }_{\Gamma_{\text{NE}}}-{\langle g_{v}(t),\psi \rangle }_{\Gamma_{\text{NE}}}=0,$$

where $u_{D}$ and $v_{D}$ are the estimated concentrations accounting for diffusion only. We then compensate for local changes in volume and surface area due to advection as in [88]:

(62) $$u(t)=\frac{J(t-\Delta t)}{J(t)}u_{D}(t),$$

(63) $$v(t)=\frac{\alpha (t-\Delta t)}{\alpha (t)}v_{D}(t).$$

The reaction terms for each surface and volume species are:

(64) $$f_{L_{\text{phos}}}=0,$$

(65) $$R_{L_{\text{phos}}}=\frac{1}{N_{\text{conv}}}\left(k_{\text{rl}}[L]-k_{\text{fl}}\frac{p_{\text{cyto}}{\left[F\right]}^{2.6}}{C_{L}+p_{\text{cyto}}{\left[F\right]}^{2.6}}{\left[L\right]}_{\text{phos}}\right),$$

(66) $$f_{Y_{\text{nuc}}}=0,$$

(67) $$R_{Y_{\text{nuc}}}=\frac{1}{N_{\text{conv}}}(k_{\text{in},\text{tot}}{\left[Y\right]}_{\text{free}}-k_{\text{out}}{\left[Y\right]}_{\text{nuc}}),$$

(68) $$g_{NPC_{A}}=k_{\text{fNPC}}[F][L]{\left[M\right]}_{A}[NPC]-k_{\text{rNPC}}{\left[NPC\right]}_{A},$$

(69) $$g_{NPC}=-k_{\text{fNPC}}[F][L]{\left[M\right]}_{A}[NPC]+k_{\text{rNPC}}{\left[NPC\right]}_{A}.$$

#### 6.2.3 Mapping species from the inner to outer NE

For lamins present at the inner NE surface, we define an additional mapping. First, we write the natural mapping from the coordinates at the outer NE reference coordinates, $X_{\text{outer}}$, to the inner NE reference coordinates $X_{\text{inner}}$:

(70) $$X_{\text{inner}}=X_{\text{center}}+(X_{\text{outer}}-X_{\text{center}})\left(\frac{R_{0}-\Delta T}{R_{0}}\right),$$

where $X_{\text{center}}=[0,0,z_{nuc}+R_{0}]$ (see Equations (2) and (3)). Then, we can express the displacement of the deformed inner surface with respect to $X_{\text{outer}}$:

(71) $$u_{\text{inner}}≔x_{\text{inner}}-X_{\text{outer}}=u[X_{\text{inner}}]+X_{\text{inner}}-X_{\text{outer}},$$

where square brackets ([⋅]) are used to indicate function evaluation at the indicated coordinates. The associated deformation gradient tensor $F_{\mathrm{alt}}$ is then defined as:

(72) $$F_{\text{alt}}≔\frac{\partial }{\partial X_{\text{outer}}}(X_{\text{outer}}+u_{\text{inner}})=\frac{\partial X_{\text{inner}}}{\partial X_{\text{outer}}}F[X_{\text{inner}}]=\left(\frac{R_{0}-\Delta T}{R_{0}}\right)F[X_{\text{inner}}],$$

The associated mapping is then:

(73) $$dx_{\text{inner}}=dX_{\text{outer}}\cdot F_{\text{alt}}^{T}.$$

Using the following relations (analogous to Equations (27) and (28)):

(74) $$\alpha_{\text{inner}}=|\;\text{det}(F_{\text{alt}})N\cdot F_{\text{alt}}^{-1}|,$$

(75) $$n_{\text{inner}}=\frac{\text{det}(F_{\text{alt}})N\cdot F_{\text{alt}}^{-1}}{\alpha_{\text{inner}}},$$

the resulting definition of the surface gradient is:

(76) $$\nabla_{S,\text{inner}}=\nabla -n_{\text{inner}}(n_{\text{inner}}\cdot \nabla )=F_{\text{alt}}^{-T}\cdot \bar{\nabla }-{\left(\frac{\text{det}\;F_{\text{alt}}}{\alpha_{\text{inner}}}\right)}^{2}(N\cdot F_{\text{alt}}^{-1})\left(\left(N\cdot F_{\text{alt}}^{-1})\cdot (F_{\text{alt}}^{-T}\cdot \bar{\nabla }\right)\right),$$

and the $L^{2}(O)$-inner product written as an integral over the reference surface $\Gamma_{\text{outer}}$ is:

(77) $${\langle a,b\rangle }_{\Gamma_{\text{inner}}}=\int_{\Gamma_{\text{outer}}}a\cdot b\alpha_{\text{inner}}d\Gamma .$$

Given these definitions, we give the weak form for dephosphorylated lamins:

(78) $${\langle \partial_{t}[L],\chi \rangle }_{\Gamma_{\text{inner}}}+{\langle D_{L}\nabla_{S,\text{inner}}[L],\nabla_{S,\text{inner}}\chi \rangle }_{\Gamma_{\text{inner}}}+{\langle [L]v_{\text{NE},\text{inner}},\nabla_{S,\text{inner}}\chi \rangle }_{\Gamma_{\text{inner}}}-{\langle g_{L},\chi \rangle }_{\Gamma_{\text{inner}}}=0,$$

where

(79) $$g_{L}=-k_{\text{rl}}[L]+k_{\text{fl}}\frac{p_{\text{cyto}}{\left[F\right]}^{2.6}}{C_{L}+p_{\text{cyto}}{\left[F\right]}^{2.6}}{\left[L\right]}_{\text{phos}},$$

and χ is a test function. In keeping with the above splitting of diffusion and advection, we solve this in two steps:

(80) $$\frac{1}{\Delta t}{\langle {\left[L\right]}_{D}(t)-[L](t-\Delta t),\chi \rangle }_{\Gamma_{\text{inner}}}+{\langle D_{L}\nabla_{S,\text{inner}}{\left[L\right]}_{D}(t),\nabla_{S,\text{inner}}\chi \rangle }_{\Gamma_{\text{inner}}}-{\langle g_{L}(t),\chi \rangle }_{\Gamma_{\text{inner}}}=0,$$

(81) $$[L](t)=\frac{\alpha_{\text{inner}}(t-\Delta t)}{\alpha_{\text{inner}}(t)}{\left[L\right]}_{D}(t).$$

#### 6.2.4 Calculation of cytosolic variables

We assume that cytosolic YAP/TAZ is well-mixed, resulting in a single concentration of free YAP/TAZ at each time. We furthermore assume that the sequestration/de-sequestration of YAP/TAZ in the cytosol is at pseudo-steady state; that is:

(82) $$(k_{\text{CN}}+k_{\text{CY}}{\left[F\right]}_{0}{\left[M\right]}_{A}){\left[Y\right]}_{\text{seq}}-k_{\text{NC}}{\left[Y\right]}_{\text{free}}=0,$$

where $k_{\mathrm{CN}}$ and $k_{\mathrm{CY}}$ are the cytoskeleton-independent and -dependent de-sequestration rates, respectively. Note that the global actin concentration ${\left[F\right]}_{0}$ is used here, as the concentration adopted in the majority of the cytosol. Assuming a conserved number of YAP/TAZ molecules over time, $N_{Y}$ and a conserved total cell volume voltot ${\mathrm{vol}}_{\mathrm{tot}}$, we can compute the total cytosolic concentration as:

(83) $${\left[Y\right]}_{\text{cyto}}=\frac{N_{Y}-N_{\text{conv}}\int_{\Omega_{\text{nuc}}}{\left[Y\right]}_{\text{nuc}}d\Omega }{N_{\text{conv}}({\text{vol}}_{\text{tot}}-\int_{\Omega_{\text{nuc}}}d\Omega )},$$

where $N_{\mathrm{conv}}$ is a conversion factor, 602.2 (molecules/μm^3^)/μM. Observing that ${\left[Y\right]}_{\mathrm{cyto}}={\left[Y\right]}_{\mathrm{free}}+{\left[Y\right]}_{\mathrm{seq}}$, we can then write ${\left[Y\right]}_{\mathrm{free}}$ at any time point:

(84) $${\left[Y\right]}_{\text{free}}={\left[Y\right]}_{\text{cyto}}\frac{k_{\text{CN}}+k_{\text{CY}}{\left[F\right]}_{0}{\left[M\right]}_{A}}{k_{\text{NC}}+k_{\text{CN}}+k_{\text{CY}}{\left[F\right]}_{0}{\left[M\right]}_{A}}.$$

Cytosolic F-actin is computed from Equation (31), which requires estimates of the distance between the PM and the NE and the local curvature of the PM. We assume a steric radius of 0.05 μm between the nanopillar and PM and consider only the closest nanopillar (centered at ($x_{\mathrm{NP}}$, $y_{\mathrm{NP}}$)) to a given point on the NE. Assuming a cylindrical PM geometry, the distance to the PM at the side and the top of the nanopillar are then given by:

(85) dtop={z+dstericifrlocal≤rNP+0.05(rlocal−rNP)2+(z+dsteric)2otherwise},

(86) dside={(rlocal−rNP)2+(z+dsteric)2ifz>−dstericrlocal−rNPotherwise},

where $r_{\mathrm{local}}=\sqrt{{\left(x-x_{\mathrm{NP}}\right)}^{2}+{\left(y-y_{\mathrm{NP}}\right)}^{2}}$ and $d_{\mathrm{steric}}$ is a gap due to steric repulsion between the PM and NE. According to the assumed geometry, PM curvature at the top is identically zero and curvature at the side is $\frac{1}{2(r_{\mathrm{NP}}+0.05)}$. Given these definitions, we solve for F-actin using Equation (31) as follows:

(87) [F]={[F]0+[F]1exp(−z+dstericdF)ifhNP=0[F]0+[F]1max[exp(−dtopdF),exp(−dsidedF)exp(12(rNP+0.05)H0)]otherwise},

where $h_{\mathrm{NP}}=0$ corresponds to a flat substrate. The use of the maximum in the second case guarantees a smooth transition in the F-actin concentration from the side to the top region of the NE near the nanopillar.

#### 6.2.5 Software implementation

We implement this coupled system using a new version of our software, Spatial Modeling Algorithms for Reactions and Transport (SMART) [89], now equipped to include advective transport. The hyperelastic problem is solved using the FEniCS Python API, allowing us to easily pass solutions to and from SMART, which also uses FEniCS. Our code is readily available on Github [90]. In brief, solving the coupled mechanochemical system involves the following steps:

1. Compute resting NE displacement (u) after applying osmotic pressure ΔP.
2. Initialize reaction-transport system in SMART over reference mesh with initial deformation u.
3. Update nuclear deformation according to stiffness governed by initial lamin content (Equation (40)).
4. Compute [F] (Equation (87)), $\alpha_{\mathrm{init}}$ (Equation (27)), and ${\left[Y\right]}_{\mathrm{free}}$ (Equation (84)) over deformed coordinates.
5. Update cap stress to $\sigma_{\text{cap}}(t+\Delta t)$ and solve for new nuclear displacement (Equation (42)). Time step is restricted such that the maximum increment in applied stress is 2 kPa.
6. Update pressure ΔP according to Equation (30) and solve for updated displacements. Repeat this process until the change in pressure is less than 1%.
7. Update [F] (Equation (31)) and α (Equation (27)).
8. Solve for updated concentrations according to reaction-diffusion (Equations (60), (61) and (80)).
9. Account for changes in local volume due to advection (Equations (62), (63) and (81)).
10. Update ${\left[Y\right]}_{\mathrm{free}}$ (Equation (84)) and NE stiffness (Equation (40)).
11. Repeat steps 5-10 until t>10000s.

### 6.3 Experimental Methods

#### 6.3.1 Nanopillar substrate fabrication and functionalization

Quartz nanopillar arrays were fabricated using projection photolithography and reactive ion etching (RIE) as previously described [17, 18, 91]. Briefly, 4-inch fused quartz wafers (Wafer Pro) were patterned using a Heidelberg MLA system and a chromium hard mask. The pattern was transferred onto the quartz substrate via REI using Ar/CHF3 plasma (Oxford Plasmalab 80), followed by wet etching to remove the mask and residual defects. The resulting nanopillars had a height of 3.24 ± 0.25 μm, diameter of 0.90 ± 0.016 μm, and a center-to-center pitch of 3.49 ± 0.06 μm.

Prior to seeding cells, nanopillar chips were sterilized and then washed with 70% ethanol and dionized (DI) water. After being air-dried, the chips underwent UV-ozone treatment for 10 min. To promote cell adhesion, the chips were functionalized with a sequential coating of 100 μg/mL poly-L-lysine (PLL) for 30 min at room temperature (R), 0.5% glutaraldehyde for 30 min (RT), and 0.1 wt% gelatin for 30 min at 37°C. After functionalization, chips were washed with DI water and stored in phosphate-buffered saline (PBS) prior to cell seeding.

#### 6.3.2 Cell culture and seeding

U2OS cells (ATCC, USA) were maintained in McCoy’s 5A (modified) Medium (ATCC, USA) supplemented with 10% fetal bovine serum (FBS, Sigma-Aldrich, USA) and 1% penicillin-streptomycin (P/S, Sigma-Aldrich, USA) at 37°C and 5% CO_2_. For experiments, cells were detached using TrypLE Express Enzyme (1X) (Gibco, USA) and resuspended to a density of 5 × 10^5^ cells/mL. A 100 μL droplet of the cell suspension (~50,000 cells) was dispensed onto the functionalized nanopillar chips in a 24-well plate. Cells were allowed to adhere for 10 min before the addition of 1 mL of growth media.

### 6.3.3 Lamin A/C silencing

To induce lamin A/C depletion, U2OS cells were transfected with lamin A/C siRNA (sc-35776, Santa Cruz Biotechnology) using the siRNA Transfection Reagent (sc-29528) and Transfection Medium (sc-36868) according to the manufacturer’s protocol. The siRNA stock was reconstituted in RNase-free water to a concentration of 10 μM prior to transfection. Knockdown efficiency was subsequently confirmed via immunofluorescence staining using lamin A/C antibodies.

#### 6.3.4 Immunofluorescence Staining

Cells were fixed with 4% paraformaldehyde (PFA, Electron Microscopy Sciences, USA) for 10 min at room temperature, washed with PBS to remove excess PFA, and then permeabilized with 1% Triton X-100 (Sigma-Aldrich, USA) for 10 min. Samples were then blocked with 2% w/v Bovine Serum Albumin (BSA, Thermo Scientific, USA) for 1 h. For primary antibody labeling, samples were incubated with mouse anti-Lamin A/C (1:400, Biolegend Cat: 600002) and rabbit anti-Ku-80 (1:200, Cell Signaling) in 1% BSA overnight at 4°C. Following PBS washes, secondary antibodies conjugated to Alexa Fluor 647 (anti-mouse) and Alexa Fluor 488 (anti-rabbit) (Thermo Fisher Scientific, USA) were applied at a 1:1000 dilution for 1 h. Following antibody incubation, samples were washed with PBS. Nuclei were stained with 4’,6-diamidino-2-phenylindole (DAPI, Thermo Scientific, USA) for 5 min.

#### 6.3.5 Fluorescence Microscopy

Fluorescence images were acquired using an Echo Revolution microscope equipped with a 60x PLAN Fluorite Water Dipping ob jective (NA = 1.00).

#### 6.3.6 Image Analysis

Image analysis was performed using ImageJ 1.53 (NIH, USA). Prior to segmentation, images were pre-processed using a rolling ball background subtraction (radius: 50 pixels, sliding parabolic enabled) and a Gaussian blur (σ=2.0) to reduce noise. Nuclear boundaries were segmented by thresholding the DAPI channel (Otsu’s method). To define the whole-cell boundary, the transmitted light channel was used to outline the cell edge. The mean fluorescence intensity (MFI) of lamin A/C was measured within the defined nuclear boundary. For Ku-80, the cytoplasmic-to-nuclear ratio (C/N) was determined via line profile analysis. A linear region of interest (ROI) was drawn across the nucleus extending into the cytoplasm. Intensity profiles were generated to capture both the nuclear signal and the perinuclear cytoplasmic plateau. The C/N was calculated by dividing the mean cytoplasmic intensity by the mean nuclear intensity after normalizing for background signal.

#### 6.3.7 Rupture Classification

NER was classified based on the Ku-80 C/N. To objectively determine the threshold separating ruptured from non-ruptured cells, a Gaussian Mixture Model (GMM) was applied to the population distribution of Ku-80 C/N. The GMM analysis identified two distinct clusters corresponding to non-ruptured and ruptured phenotypes. The intersection point of the fitted Gaussian distributions provided a data-driven threshold of 0.16. Cells with a Ku-80 C/N exceeding this value were classified as ruptured.

#### 6.3.8 Statistical Analysis

All experimental results were taken from at least three independent experiments. Statistical comparisons shown in Figure 7 were performed using unpaired Student’s *t*-tests in GraphPad Prism software.

## Supplementary Material

Supplement 1 **Movie 1:** NE stretch (α) during nuclear compression withs $\sigma_{\text{cap}}=0-1000$ Pa on flat versus nanopillar substrates. This movie shows a flat substrate, followed by substrates with $r_{\text{NP}}=200\;\text{nm}$, $h_{\text{NP}}=1.5\;\mu m$, and $p_{\text{NP}}=2\;\mu m$, 4 μm, 5 μm, and 6 μm. The final section shows all pitches simultaneously for comparison.
**Movie 2:** NE tension (γ) during nuclear compression with $\sigma_{\text{cap}}=0-1000$ Pa on flat versus nanopillar substrates. This movie shows a flat substrate, followed by substrates with $r_{\text{NP}}=200\;\text{nm}$, $h_{\text{NP}}=1.5\;\mu m$, and $p_{\text{NP}}=2\;\mu m$, 4 μm, 5 μm, and 6 μm. The final section shows all pitches simultaneously for comparison.
**Movie 3:** NPC activation on the outer surface of the NE during simulations with $\sigma_{\max}=200$ Pa versus $\sigma_{\max}=800f$ Pa on flat and nanopillar ($r_{\text{NP}}=200\;\text{nm}$, $h_{\text{NP}}=1.5\;\mu m$, and $p_{\text{NP}}=3\;\mu m$) substrates. $t_{0,\text{cap}}=1000\;s$ in all cases shown.
**Movie 4:** Lamin density at the inner surface of wild-type nuclei during compression on a nanopillar substrate ($r_{\text{NP}}=200\;\text{nm}$, $h_{\text{NP}}=1.5\;\mu m$, and $p_{\text{NP}}=5\;\mu m$) for faster $\left(t_{0,\text{cap}}=100\;s\right)$ versus slower $\left(t_{0,\text{cap}}=1000\;s\right)$ rates of cap assembly. $\sigma_{\max}=400$ Pa in the cases shown.
**Movie 5:** Force per lamin subunit at the inner surface of wild-type nuclei during compression on a nanopillar substrate ($r_{\text{NP}}=200\;\text{nm}$, $h_{\text{NP}}=1.5\;\mu m$, and $p_{\text{NP}}=5\;\mu m$) for faster $t_{0,\text{cap}}=1000\;s)$ versus slower $t_{0,\text{cap}}=1000\;s)$ rates of cap assembly. $\sigma_{\max}=400$ Pa in the cases shown.
**Movie 6:** Lamin density at the inner surface of low-lamin (50% expression) nuclei during compression on a nanopillar substrate ($r_{\text{NP}}=200\;\text{nm}$, $h_{\text{NP}}=1.5\;\mu m$, and $p_{\text{NP}}=5\;\mu m$) for faster $t_{0,\text{cap}}=1000\;s)$ versus slower $t_{0,\text{cap}}=1000\;s)$ rates of cap assembly. $\sigma_{\max}=400$ Pa in the cases shown.
**Movie 7:** Force per lamin at the inner surface of low-lamin (50% expression) nuclei during compression on a nanopillar substrate ($r_{\text{NP}}=200\;\text{nm}$, $h_{\text{NP}}=1.5\;\mu m$, and $p_{\text{NP}}=5\;\mu m$) for faster $t_{0,\text{cap}}=1000\;s)$ versus slower $t_{0,\text{cap}}=1000\;s)$ rates of cap assembly. $\sigma_{\max}=400$ Pa in the cases shown.

Supplement 2

## Figures and Tables

**Table 1:** Parameters governing nuclear mechanics.

	Value	Description	Sources/Notes
$R_{0}$	4.1 μm	Stress-free nuclear radius	5.0 μm post-inflation [74]*
ΔT	0.2 μm	Stress-free NE thickness	0.13 μm post-inflation [75]**
$z_{\text{nuc}}$	1.04 μm	Initial location of the NE bottom	Ensures substrate contact post-inflation
$Z_{0}$	2.05 μm	Characteristic decay length for cap stress	Restricts to mainly upper half of NE
$P_{\max}$	820 Pa	Pressure difference across the NE	[33, 34]
$E_{1}$	5000 Pa	First Mooney-Rivlin elastic constant	[34]
$E_{2}$	1000 Pa	Second Mooney-Rivlin elastic constant	[34]
$t_{0,\text{cap}}$	100-1000 s	Time scale for perinuclear actin cap formation	Varied for testing
$\sigma_{\max}$	0-800 Pa	Maximum perinuclear actomyosin force	Varied for testing
$\phi_{\text{inner}}$	100 Pa	Nucleoplasm bulk modulus	Constrained by volume loss [14, 33]
$\phi_{0}$	1 × 10^8^ Pa	Bulk modulus (incompressibility constraint)	Set by numerical testing
$\sigma_{\text{contact},0}$	1000 Pa	Contact repulsion strength	Set by numerical testing
$d_{\text{steric}}$	0.2 μm	Steric repulsion radius	Set by numerical testing
$h_{\text{edge}}$	0.15 μm	Mesh resolution on flat substrate	Set by numerical testing
	0.1 μm	Mesh resolution on 200 nm nanopillars	Set by numerical testing
	0.15 μm	Mesh resolution on 500 nm nanopillars	Set by numerical testing
$D_{\text{roof}}$	$10h_{\text{edge}}$	Upper region cutoff (Equation (48))	Set by numerical testing
$E_{1,\text{nuc}}$	5000 Pa	Elastic modulus for nucleoplasm deformation	Set by numerical testing
$\phi_{\text{nuc}}$	1 × 10^5^ Pa	Bulk modulus for nucleoplasm deformation	Set by numerical testing
$\Delta \hat{z}$	50 nm	Double bilayer plus perinuclear space thickness	[52]

* Post-inflation radius matches nuclear size from [74]

** Post-inflation thickness is at upper end of double membrane plus laminar thickness [75]

**Table 2:** Reaction-transport parameters.

	Value	Description	Source(s)
$d_{F}$	0.5 μm	Characteristic decay length for F-actin	*
$H_{0}$	2.0 μm	Sensitivity of actin polymerization to PM curvature	[18, 29]
${\left[F\right]}_{0}$	140 μM	Baseline actin polymerization (flat substrate)	[18, 29]
	100 μM	Baseline actin polymerization $r_{\text{NP}}=0.2\;\mu m$	[18, 29]
	120 μM	Baseline actin polymerization $r_{\text{NP}}=0.5\;\mu m$	[18, 29]
${\left[F\right]}_{1}$	20 μM	PM curvature-dependent actin polymerization	[18, 81]
$D_{L}$	0.001 μm^2^ s^−1^	NE lamin diffusion coefficient	[80]
$D_{L,\text{vol}}$	4.0 μm^2^ s^−1^	Nucleoplasmic lamin diffusion coefficient	[82]**
$k_{\text{rl}}$	0.001 s^−1^	Lamin phosphorylation rate	[80, 83]
$k_{\text{fl}}$	415.5 μm^−2^μM^−1^s^−1^	Maximum lamin dephosphorylation rate	[25, 80]***
$p_{\text{cyto}}$	9 × 10^−6^ kPa μM^−2.6^	Cytoplasmic stiffness coefficient	[80, 84]
$C_{L}$	100 kPa	Cytoplasmic stiffness sensitivity (lamin A dephos.)	[25, 80]
$D_{\text{NPC}}$	0.001	NPC diffusion coefficient	[80, 85]
$k_{\text{fNPC}}$	2.8 × 10^−7^ μm^2^ s^−1^ μM^−1^	NPC activation rate	[80]
$k_{\text{rNPC}}$	8.7 s^−1^	NPC deactivation rate	[80]
$k_{\text{in},b}$	1.0 μm^−2^ s^−1^ μM^−1^	Baseline YAP/TAZ import rate	[80]
$k_{\text{in}}$	10.0 μM s^−1^	Lamin-dependent YAP/TAZ import through NPCs	[80]
$\alpha_{0}$	5.0	NPC stretch sensitivity	[18]
$D_{Y}$	4.0 μm^2^ s^−1^	YAP/TAZ diffusion rate in nucleus	[76]
$k_{\text{CN}}$	0.56 s^−1^	Baseline YAP/TAZ activation rate	[80]
$k_{\text{CY}}$	7.6 × 10^−4^ μM^−2^ s^−1^	Actomyosin-dependent YAP/TAZ activation	[80]
$k_{\text{NC}}$	0.14 s^−1^	YAP/TAZ sequestration rate	[80]
${\text{vol}}_{\text{tot}}$	1024.0 μm^3^	Total volume for U2OS (quarter cell)	[18]
$N_{\text{NPC}}$	594.7	Total number of NPCs	[18, 80]
$N_{L}$	3.202 × 10^5^	Total number of lamin A/C subunits (wild type)	[18, 80]
	1.601 × 10^5^	Total number of lamin A/C subunits (low lamin)	[18, 80]
$N_{Y}$	5.339 × 10^5^	Total number of YAP/TAZ molecules	[18, 80]
$\phi_{\text{scale}}$	0.2	Scale factor for YAP/TAZ influx	[14]^†^
$\phi_{L}$	0.5	Relative contribution of lamin A/C to NE stiffness	[23]
${\left[L\right]}_{\text{ref}}$	2980 μm^−2^	Lamin stiffness normalization (matches WT density)	
$N_{\text{conv}}$	602.2 # μm^−3^ μM^−1^	Conversion factor	
Initial conditions (determined by mechanical model):			
${\text{SA}}_{\text{NE},\text{inner},0}$	107.5 μm^2^	Initial surface area of inner NE	
${\text{SA}}_{\text{NE},\text{outer},0}$	118.4 μm^2^	Initial surface area of outer NE	
${\text{vol}}_{\text{nuc},0}$	152.0 μm^3^	Initial volume of nucleus	
${\left[L\right]}_{\text{phos},\text{init}}=\frac{N_{L}k_{\text{rl}}}{k_{\text{fl}}{\text{vol}}_{\text{nuc},0}N_{\text{conv}}+k_{\text{rl}}{\text{SA}}_{\text{NE},\text{inner},0}}$			(SS)^††^
${\left[L\right]}_{\text{init}}=\frac{N_{L}-{\left[L\right]}_{\text{phos},\text{init}}{\text{vol}}_{\text{nuc},0}N_{\text{conv}}}{{\text{SA}}_{\text{NE},\text{inner},0}}$			(SS)^††^
${\left[NPC\right]}_{A,\text{init}}=\frac{N_{\text{NPC}}}{SA_{\text{NE},\text{outer},0}}\frac{k_{\text{fNPC}}{\left[L\right]}_{\text{init}}[F]{\left[M\right]}_{A}}{k_{\text{fNPC}}{\left[L\right]}_{\text{init}}[F]{\left[M\right]}_{A}+k_{\text{rNPC}}}$			(SS)^††^
${\left[NPC\right]}_{\text{init}}=\frac{N_{\text{NPC}}}{SA_{\text{NE},\text{outer},0}}-{\left[NPC\right]}_{A,\text{init}}$			(SS)^††^
${\left[Y\right]}_{\text{nuc},\text{init}}=\frac{N_{Y}}{N_{\text{conv}}}\frac{\frac{{\left[Y\right]}_{\text{free}}}{{\left[Y\right]}_{\text{cyto}}}\phi_{\text{scale}}\;(k_{\text{inb}}+k_{\text{in}}{\left[NPC\right]}_{A,\text{init}})}{k_{\text{out}}({\text{vol}}_{\text{tot}}-{\text{vol}}_{\text{nuc},0})+\frac{{\left[Y\right]}_{\text{free}}}{{\left[Y\right]}_{\text{cyto}}}\phi_{\text{scale}}\;(k_{\text{inb}}+k_{\text{in}}{\left[NPC\right]}_{A,\text{init}})\;{\text{vol}}_{\text{nuc},0}}$			(SS)^†††^

* Set to approximately match length scale from our previous simulations [18]

** Intermediate value used between two mobile populations of lamin A in the nucleoplasm in [82].

*** Converted from $k_{\text{fl}}$ in previous studies assuming nuclear volume-to-surface-area ratio of 1.5 μm

† Set to match range of YAP/TAZ N/C measured in [14]

†† Expressions given for predicted steady-state values in well-mixed limit.

††† Well-mixed steady-state expression uses $\frac{{\left[Y\right]}_{\text{free}}}{{\left[Y\right]}_{\text{cyto}}}$ from Equation (84).
