# Supplementary material for: A predictive mechanochemical modeling framework for the deformation and remodeling of the nuclear lamina
Source: bioRxiv. 2026 Mar 16:2026.02.19.706840. Originally published 2026 Feb 20. Preprint. [Version 2] doi: 10.64898/2026.02.19.706840 (PMC12934953; doi:10.64898/2026.02.19.706840)
Supplement: Supplement 2 [file nihpp2026.02.19.706840v2-supplement-2.pdf]

bioRxiv preprint doi: https://doi.org/10.64898/2026.02.19.706840; this version posted March 17, 2026. The copyright holder for this preprint (which was not certified by peer review) is the author/funder, who has granted bioRxiv a license to display the preprint in perpetuity. It is made available under aCC-BY 4.0 International license.

S1 Supplementary Figures

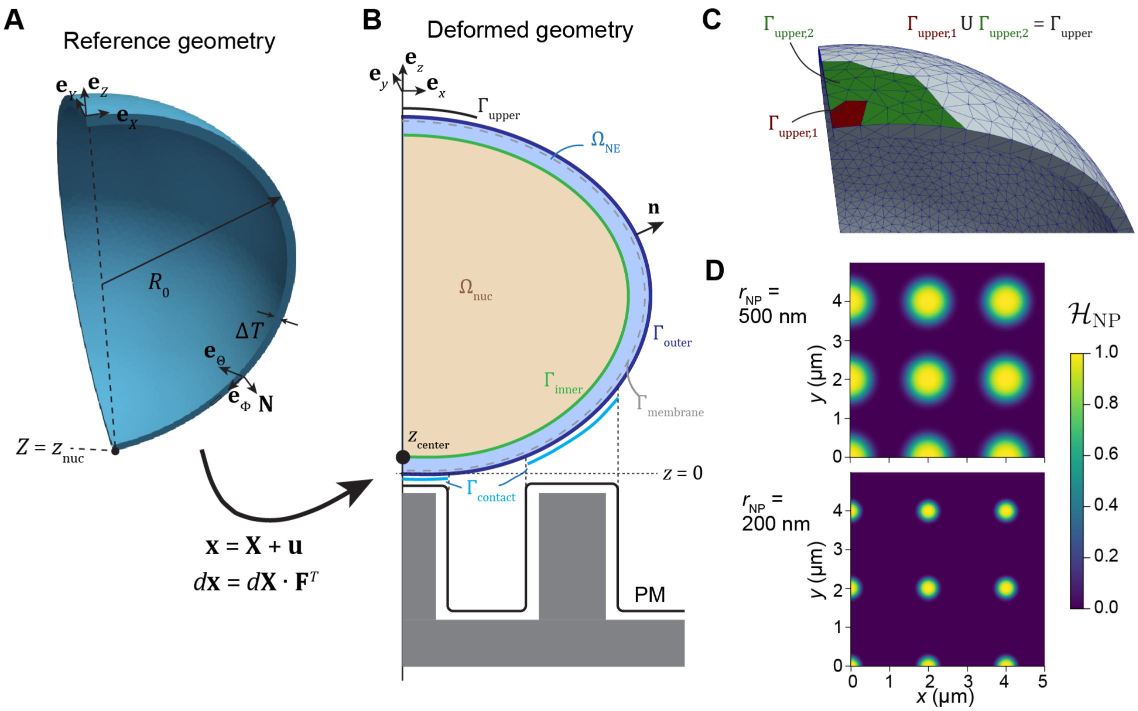

Figure S1: Coordinate and domain definitions for mechanical model. A) Reference geometry with unit vectors denoting Cartesian coordinates and spherical coordinates. B) Schematic showing volumetric domains (Ω_{nuc} and Ω_{NE}) and surface domains (Γ_{inner} and Γ_{outer}) of the nucleus. The outer NE includes subdomains Γ_{upper} and Γ_{contact} as indicated. Γ_{membrane} denotes the midplane of the nuclear membranes and perinuclear space, assumed to occupy a thickness of 50 nm in the resting configuration [52]. PM surface included for illustrative purposes. C) Depiction of upper NE subdomains included in Equation (47) and Equation (48). D) Smooth approximation of Γ_{contact} given by Equation (45) for $r_{\text{NP}} = 200$ or 500 nm, $p_{\text{NP}} = 2$ μm.

bioRxiv preprint doi: https://doi.org/10.64898/2026.02.19.706840; this version posted March 17, 2026. The copyright holder for this preprint (which was not certified by peer review) is the author/funder, who has granted bioRxiv a license to display the preprint in perpetuity. It is made available under aCC-BY 4.0 International license.

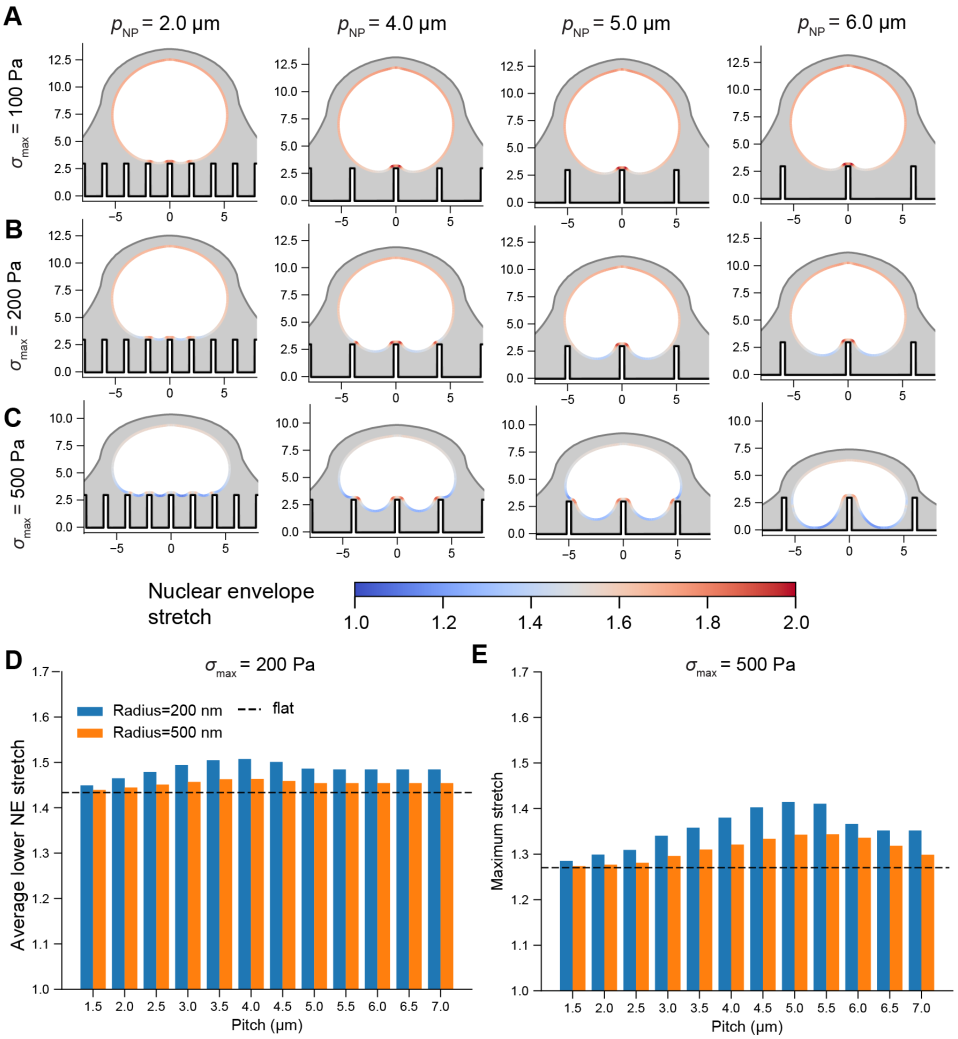

Figure S2: NE stretch on 3 µm tall nanopillars. A-C) Cross sections of equilibrium conformations of deformed nuclei on substrates with r_{NP} =200 nm, h_{NP} =3.0 µm, and p_{NP} = 2 µm, 4 µm, 5 µm, or 6 µm for cap stress (σ_{cap}) of 100 Pa (A), 200 Pa (B), or 500 Pa (C). Reported cap stresses correspond to values at the top of the nucleus. The computed stretch corresponds to stretch of the inner surface of the NE. D-E) Maximum stretch of the NE as a function of nanopillar pitch for an applied cap stress of 200 Pa (D) or 500 Pa (E) on 200 nm or 500 nm radius nanopillars. The dashed lines indicate stretch on a flat substrate.

bioRxiv preprint doi: https://doi.org/10.64898/2026.02.19.706840; this version posted March 17, 2026. The copyright holder for this preprint (which was not certified by peer review) is the author/funder, who has granted bioRxiv a license to display the preprint in perpetuity. It is made available under aCC-BY 4.0 International license.

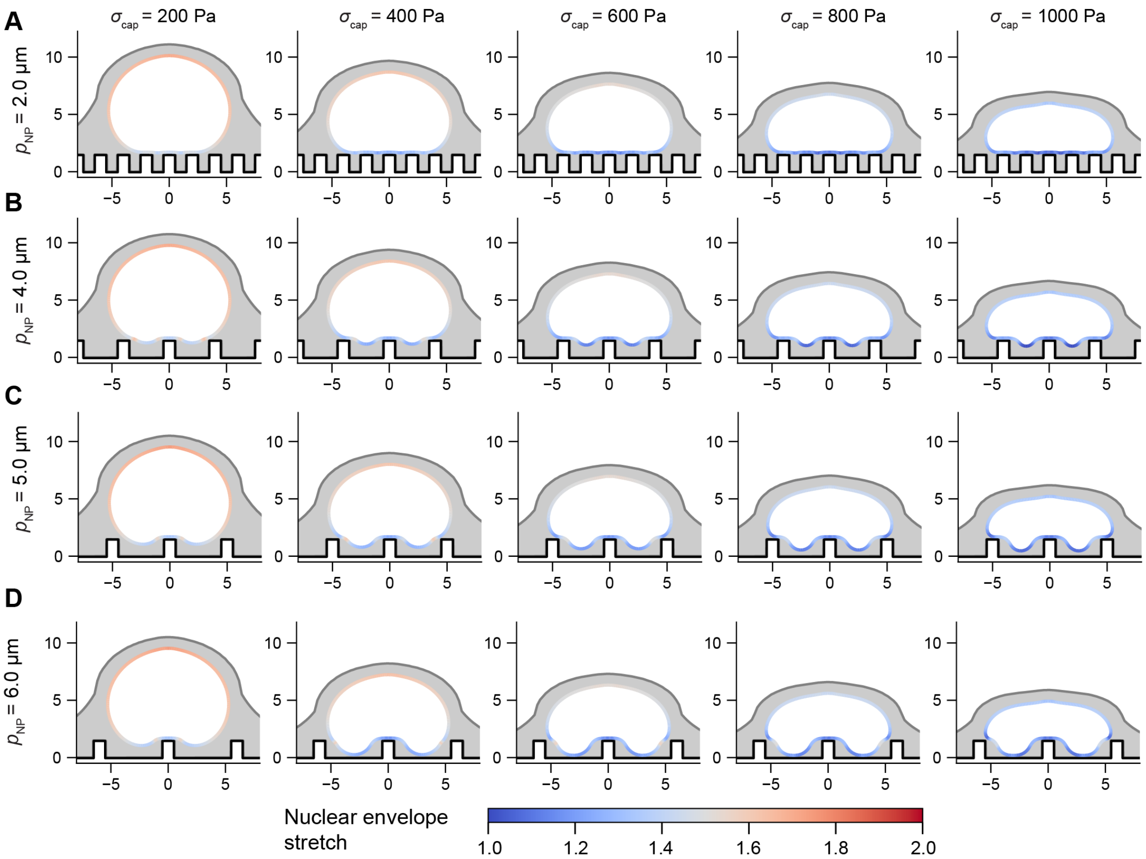

Figure S3: NE stretch on 500 nm radius nanopillars. Dynamics of nuclear compression are shown for nuclei experiencing between 200-1000 Pa cap stress on substrates with $p_{\rm NP} = 2$ µm (A), 4 µm (B), 5 µm (C), and 6 µm (D).

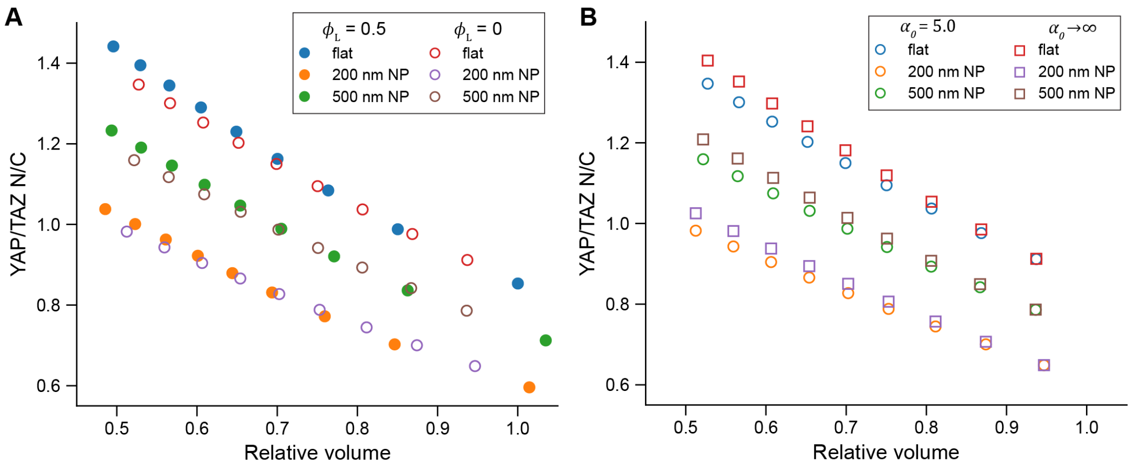

Figure S4: Correlation between YAP/TAZ and nuclear volume is consistent across different model assumptions. A) Predicted correlation between YAP/TAZ and relative volume for $\phi_{\rm L} = 0.5$ (filled circles, bidirectional coupling) and $\phi_{\rm L} = 0$ (open circles, no bidirectional coupling). B) Predicted correlation between YAP/TAZ and relative volume for $\alpha_0 = 5.0$ (open circles, NPC stretch sensitivity) and $\alpha_0 \to \infty$(open squares, no NPC stretch sensitivity). Both cases in A include NPC stretch sensitivity ($\alpha_0 = 5.0$) and both cases in B neglect bidirectional coupling ($\phi_{\rm L} = 0$). All volumes are normalized to nuclear volume on a flat substrate with no applied force for $\alpha_0 = 5.0$, $\phi_{\rm L} = 0.5$.
